# Combination of Systems Pharmacology and Experimental Evaluation to Explore the Mechanism of Synergistic Action of Frankincense-Myrrh in the Treatment of Cerebrovascular Diseases

**DOI:** 10.3389/fphar.2021.796224

**Published:** 2022-01-10

**Authors:** Yucheng Liao, Jingwen Wang, Chao Guo, Min Bai, Bowei Ju, Zheng Ran, Junping Hu, Jianhua Yang, Aidong Wen, Yi Ding

**Affiliations:** ^1^ College of Pharmacy, Xinjiang Medical University, Urumqi, China; ^2^ Department of Pharmacy, Xijing Hospital, Fourth Military Medical University, Xi’an, China; ^3^ Department of Pharmacy, The First Affiliated Hospital, Xinjiang Medical University, Urumqi, China

**Keywords:** frankincense-myrrh, cerebrovascular disease, systems pharmacology, experimental evaluation, synergistic effect

## Abstract

Frankincense-Myrrh is a classic drug pair that promotes blood circulation, and eliminates blood stasis. The combination of the two drugs has a definite clinical effect on the treatment of cerebrovascular diseases (CBVDs), but its mechanism of action and compatibility have not been elucidated. In this study, the bioactive components, core targets, and possible synergistic mechanisms of Frankincense-Myrrh in the treatment of CBVDs are explored through systems pharmacology combined with *in vivo* and *in vitro* experiments. Comparing target genes of components in Frankincense and Myrrh with CBVD-related genes, common genes were identified; 15 core target genes of Frankincense-Myrrh for the treatment of CBVDs were then identified using protein-protein interaction (PPI) analysis. It was also predicted through Gene Ontology (GO) and Kyoto Encyclopedia of Genes and Genomes (KEGG) pathway enrichment analysis that the molecular mechanism of Frankincense-Myrrh action on CBVDs was mainly related to the regulation of neurotrophic factors and inflammatory responses. Frankincense-Myrrh significantly improved neurological function, decreased infarct volume, alleviated histopathological damage, inhibited microglial expression, and promoted the expression of neurons in middle cerebral artery occlusion (MCAO)-induced rats. The results of this study not only provide important theoretical support and experimental basis for the synergistic effect of Frankincense-Myrrh, but also provide new ideas for the prevention and treatment of cerebral ischemic injuries.

## Introduction

Cerebrovascular diseases (CBVDs) including ischemic stroke are characterized by high morbidity, mortality, and disability, and seriously threaten human health and quality of life (Feng et al., 2019; Karschnia et al., 2019). Currently, treatment is mainly focused on early thrombolysis to restore protective cerebral flow, and achieve vascular recanalization (Liu C et al., 2021). However, most patients cannot receive thrombolytic therapy because of limitations in the treatment time window, or other contraindications (Damani et al., 2018). Traditional Chinese medicine (TCM) has accumulated rich experience in the treatment of stroke, and has achieved positive clinical effects (Sun et al., 2015; Bu et al., 2020; Wang et al., 2020). TCM believes that blood stasis blocking the brain collaterals is the core pathology of stroke. Therefore, drugs for promoting blood circulation and removing blood stasis are commonly used in TCM to treat strokes, to dissipate blood stasis in the body, regenerate new blood, and unblock blood vessels (Chan et al., 2018; Wang C et al., 2018). This is similar to the “vascular recanalization and angiogenesis” in modern medicine.

Frankincense-Myrrh is a classic medicine pair that promotes blood circulation and removes blood stasis. The combination of the two drugs has a definite clinical effect on the prevention and treatment of stroke (Forouzanfar et al., 2016; Baram et al., 2019; Cao et al., 2019). However, the molecular mechanisms underlying cerebrovascular protection have not yet been elucidated, and the compatibility of its active ingredients has rarely been reported. Our previous studies have confirmed that the active ingredient acetyl-11-keto-β-boswellic acid (AKBA) in Frankincense can significantly reduce the area of cerebral infarction (Ding et al., 2014; Ding et al., 2015). Z-Guggulsterone (Z-GS) in myrrh has a significant effect in resisting ischemic brain tissue damage, and can also significantly reduce the area of cerebral infarction (Liu et al., 2020). The application of modern science and technology to clarify the effective ingredients and signal network of Frankincense-Myrrh for synergistic action for brain protection requires urgent attention.

Systems pharmacology provides a good opportunity for the modernization of Chinese medicine, especially the modernization of its theoretical basis (Liao et al., 2021; Liu N et al., 2021). Systems pharmacology can quickly clarify the mechanism of multi-components in TCM that play an overall role through multiple targets and channels (Yang et al., 2017; Qin et al., 2021; Xu et al., 2021). The panoramic view it provides and its comprehensive nature—combined with the multi-component characteristics and integration of TCM compounds—offers a holistic view of TCM theory, and the treatment based on syndrome differentiation (Pan et al., 2020). A number of studies based on systems pharmacology have successfully revealed the working of TCM in the treatment of CBVDs, such as Erigeron breviscapus (Wang J et al., 2018), Shuxuening injections (Cui et al., 2020), and Guanxin-Shutong capsule (Zhang et al., 2021).

**FIGURE 1:**
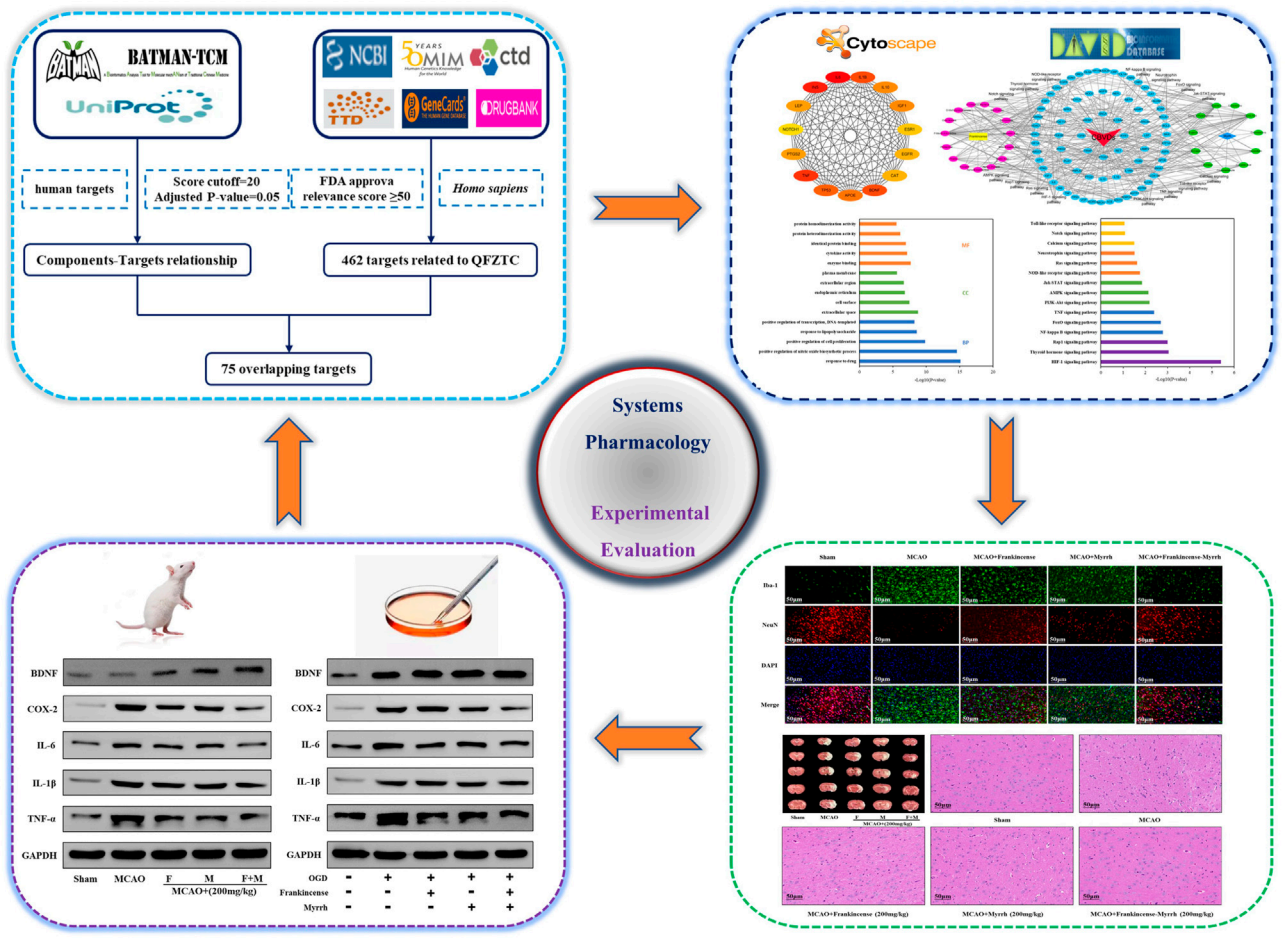
Workflow of systems pharmacology analysis and experimental evaluation.

This study combined systems pharmacology and experimental evaluation to explore the synergistic protective mechanism of Frankincense-Myrrh against CBVDs. The detailed workflow is shown in Figure 1. First, a systems pharmacology method was used to predict the effective components and core targets of Frankincense-Myrrh in the treatment of CBVDs, and for network construction and enrichment analysis. Subsequently, an animal model of middle cerebral artery occlusion (MCAO) was established to study the synergistic therapeutic effects of Frankincense-Myrrh in treating ischemic stroke, and to explore the molecular mechanism of its action against CBVDs. Finally, the expression of core targets was verified by subjecting brain microvascular endothelial cells (BMECs) to oxygen-glucose deprivation (OGD) in order to systematically investigate the potential interactions between bioactive components, key targets, and pathways.

## Materials and Methods

### Plant Materials

Frankincense (*Boswellia sacra* Flück) and Myrrh (*Commiphora myrrha* (T.Nees) Engl) were supplied by Sinuote Bio-Technology (Shaanxi, China). All herbs were identified by Professor Aidong Wen, deposited at the Fourth Military Medical University, and met Chinese Pharmacopoeia requirements (2020 Edition). Respectively take 50 g of raw drug was dissolved in 100 ml of 75% ethanol ground into powder (1 mm mesh), and extracted for 120 min at 80°C, three consecutive times. Then the alcohol extract was filtered (centrifuge for 10 min, 3200 rcf). The supernatants were harvested and vacuum-dried to obtain powdered samples (10g, 1 g contains 5 g raw drug).

### Chemicals and Reagents

Goat anti-rabbit IgG (98164S), rabbit anti-Iba-1 (17198S), rabbit anti-NeuN (24307S), rabbit anti-COX2 (12282S), and rabbit anti-GAPDH (2118S) were obtained from Cell Signaling Technology (Danvers, MA, United States). Rabbit anti-BDNF (ab108319), rabbit anti-IL6 (ab259341), rabbit anti-IL1β (ab254360), and rabbit anti-TNF-α (ab205587) were purchased from Abcam (Cambridge, MA, United States). 2,3,5-triphenyltetrazolium chloride (TTC, purity >98.0%) was purchased from Sigma-Aldrich (St. Louis, MO, United States). Sodium dodecyl sulfate-polyacrylamide gel electrophoresis (SDS-PAGE) and bicinchoninic acid (BCA) protein assay kits were purchased from Dingguo Changsheng Biotechnology (Beijing, China). Tris-buffered saline (TBS), transfer buffer (pH 8.4), and running buffer (pH 8.4–8.6) were purchased from Servicebio (Wuhan, China). Other reagents were provided by other suppliers.

### Identification of Components and Targets of Frankincense-Myrrh

Components and targets of Frankincense-Myrrh were identified through a key-word search using the words “Ruxiang” and “Moyao” in the Herb or Herb list, followed by the selection of a protein with high reliability as the target (score cutoff = 20, adjusted *p*-value = 0.05) from the BATMAN-TCM database^1^, a bioinformatics evaluation tool for online predictions of the molecular mechanisms of drugs used in TCM (Liu et al., 2016). All the targets thus obtained were imported to the UniProt database^2^, non-human targets were removed to isolate relevant gene names, and all target information related to active components were recorded.

### Identification of Target Genes of Cerebrovascular Diseases

To clarify the relationship between targets and diseases from different perspectives, the target genes related to CBVDs were taken from six databases. “cerebrovascular” or “cerebrovascular diseases” was used as the keyword to search the NCBI Gene^3^, OMIM^4^, CTD^5^, TTD^6^, GeneCards^7^, and Drugbank^8^ databases. To ensure the accuracy of target identification, we only selected targets with an inference score ≥50 in the CTD database, relevance score ≥50 on the GeneCards website, and FDA-approved targets on the DrugBank website. After removing duplicates, candidate targets associated with CBVDs were identified.

### Protein-Protein Interaction

The Frankincense-Myrrh targets and the targets related to CBVDs were imported to the Venny2.1 website^9^ to obtain the targets common to drug components and diseases. The common targets were imported to the STRING database^10^ to construct a protein-protein interaction network model; the protein type was set to “Homo sapiens,” and the confidence score to greater than 0.7, keeping other parameters at the default settings, to obtain the PPI network.

### Network Construction and Enrichment Analysis

The common targets were imported to the Cytoscape 3.7.1 software to construct a visual network diagram of the targets of Frankincense-Myrrh in the treatment of CBVDs. GO enrichment analysis was performed using the DAVID online analysis tool. When *p* < 0.05, the results of the GO enrichment analysis were considered statistically significant, and the functional mechanisms as necessary. The DAVID database was used for KEGG pathway enrichment analysis to identify the specific signaling pathway of Frankincense-Myrrh in the treatment of CBVDs.

### Design of *in vitro* Experiment

BMECs from rats were cultured *in vitro* and divided into five groups: control group (Control), model group (OGD), OGD + Frankincense (10 μM), OGD + Myrrh (10 μM) and OGD + Frankincense-Myrrh (10 μM); The model group and administration groups were subjected to OGD modeling. The corresponding drugs were added 30 min before hypoglycemia and hypoxia set in. Except for the control group, the groups were cultured for 48 h under normal conditions, after 6 h of OGD treatment.

### Cultivation of Brain Microvascular Endothelial Cells

The cerebral cortex of the rat brain was separated. After rinsing with DMEM, it was subjected to type II collagenase digestion, centrifugation, and inoculation in a culture flask coated with gelatin. The culture medium was DMEM. Primary cultures of BMECs were carried out in an incubator. After the cells grew into a monolayer, they were digested and passaged, the culture conditions being the same as above. The BMECs were morphologically identified, and the immunohistochemical factor VIII-related antigen test was confirmed to be positive; the third generation BMECs were then gathered for the experiment.

### Establishment of Oxygen-Glucose Deprivation Model

After the cell culture matured, the cell culture medium was discarded, the cells were washed with PBS three times, PBS was added as the new culture medium, and the culture placed in a 37°C incubator containing 95% N_2_ + 5% CO_2_ for 6 h. The culture medium was then replaced, and the culture incubated at 37°C in a 5% CO_2_ incubator to restore the supply of glucose and oxygen.

### Design of Experiment Involving Animals

A total of 60 male Sprague-Dawley (SD) rats (250–280 g) aged 8 weeks were obtained from the Experimental Animal Center of the Fourth Military Medical University. Under light conditions alternating between 12/12 h, the rats were placed in an environment with an indoor humidity of 45–75% and temperature of 22 ± 2°C, and provided free access to water and food. Rats were acclimatized to laboratory conditions for ten days before establishing the MCAO model. The experiment was conducted with the approval of the China Food and Drug Administration (cFDA). Every effort was made to minimize the number of animals used and their suffering. Rats were randomly divided into five groups (*n* = 12 in each group): sham operation group (Sham), model group (MCAO), MCAO + Frankincense (200 mg/kg of body weight (kg BW) per day), MCAO + Myrrh (200 mg/kg BW per day), and MCAO + Frankincense-Myrrh (200 mg/kg BW per day), which was dissolved in 0.9% saline (the ratio of Frankincense-Myrrh is 1:1). Rats in the sham and MCAO groups were treated with intragastrical administration of 0.9% saline solution as vehicle control. The administration was lasted for seven days (Figure 5A). The rats were then anesthetized, and the MCAO model established. 2 h after MCAO establishment, the filament was withdrawn to allow reperfusion for 24 h. In the sham group, the same operation was performed, except that the filament was not inserted into the internal carotid artery. In this experiment, no animals died in the MCAO model. According to the experimental scheme, three of the rats in each group were used to perform TTC staining; three rats were used to perform H&E staining; three rats were used to perform western blotting; and three rats were used to perform immunofluorescence staining.

### Establishment of Middle Cerebral Artery Occlusion Model

Rats were anesthetized by intraperitoneal injection of 1% sodium pentobarbital (40 mg/kg BW); the rats were fixed in the supine position on the operating table, and the neck was clipped and disinfected. An incision approximately 2 cm in length was made to the right of the neck, and the right side was bluntly separated layer by layer, i.e., the common carotid artery, external carotid artery, and internal carotid artery. A monofilament nylon thread (0.22–0.24 mm in diameter) was inserted through the common carotid artery near the bifurcation incision, and the thread plug was inserted through the internal carotid artery to reach the circle of Willis to achieve middle cerebral artery embolization. Ligation was done with a nylon thread, and 2 h later, reperfusion was performed.

### Neurological Deficit Score

The Zea-Longa scoring method was used to score neurological deficits. The neurological deficits of rats were scored based on the following five levels: 0 points, no neurological deficit; 1 point: the left forefoot cannot be fully straightened when lifted vertically; 2 points: rearward movement of the body to the left when walking; 3 points: the body falls to the left while walking; 4 points: unable to crawl, and unconscious. Rats that died, or scored 0 or 4 during the experiment were not tested further.

### TTC Staining Method

After the rat’s brain was removed, it was placed in a refrigerator at 4°C for about 10 min; the coronal section was then evenly sliced into approximately 2 mm thick slices, and the brain slices were placed in 2% 2,3,5-triphenyltetrahydrochloride (TTC) solution at 37°C for 30 min, it was fixed with 4% paraformaldehyde buffer, and a photograph taken with a digital camera. The area stained pink was the normal brain tissue, and the white area was the infarct area. Photoshop image processing software was used to calculate infarct size.

### H&E Staining Method

To assess histopathological damage, the rats were deeply anesthetized, and euthanized 24 h after MCAO modeling. Subsequently, the rat brain tissue was quickly removed and immediately immersed in 4% paraformaldehyde for fixation for 24 h. After dehydration, transparency, wax immersion, embedding, and other operations, a wax block was made, and a coronal section (5 μm) was performed. Finally, the slides were stained with hematoxylin and eosin (H&E), and histopathological changes in the brain were observed using an optical microscope.

### Western Blot Analysis

Total proteins were extracted from ischemic penumbra tissues and BMECs subjected to OGD, using RIPA lysis buffer, and the concentrations were determined using a bicinchoninic acid (BCA) protein assay kit. Agarose gel electrophoresis was performed using the transfer membrane, including blocking with 5% skimmed milk powder for 30 min, addition of primary antibodies (1:1,000 dilution), overnight incubation at 4°C, rinsing thrice with TBST for 10 min each, addition of secondary antibodies (1:5,000 dilution), incubation at room temperature for 1 h, rinsing thrice with TBST for 10 min each, followed by observation of chemiluminescent color development and ECL kit display protein bands, gel imager imaging, taking pictures, and analyzing the band intensity using Image ProPlus 6.0 image processing software, in that order.

### Immunofluorescence

The preserved tissue pieces were deparaffinized and rehydrated, antigen in citric acid buffer were restored, the tissue pieces were naturally cooled to room temperature and blocked with 5% BSA at room temperature for 2 h, and the diluted primary antibodies incubated at 4°C overnight; the tissue was removed the next day and rewarmed for 40 min, and washed with PBS thrice for 10 min each. After incubating the secondary antibodies for 2 h at room temperature (dark operation), the samples were washed thrice with PBS for 10 min each, after which DAPI stained the nucleus for 8 min. The slides were again washed thrice with PBS for 10 min each, and finally, the anti-quenching agent was added and the slide mounted. The results were observed using a fluorescent inverted microscope.

### Statistical Analysis

Data were recorded as the mean ± standard deviation (SD). One-way analysis of variance (ANOVA) was used to examine the change in value to verify its importance. The differences between groups were analyzed by ANOVA and the Bonferroni post-hoc test. The data were analyzed using SPSS (IBM SPSS Statistics v19.0), and all statistical analyses were performed using GraphPad Prism 6.0. *p* < 0.05 was considered as statistical significance, *p* < 0.01 or *p* < 0.001 was considered as significant statistical significance.

## Results

### Component-Target Network Involving Frankincense, Myrrh, and Cerebrovascular Diseases

**FIGURE 2:**
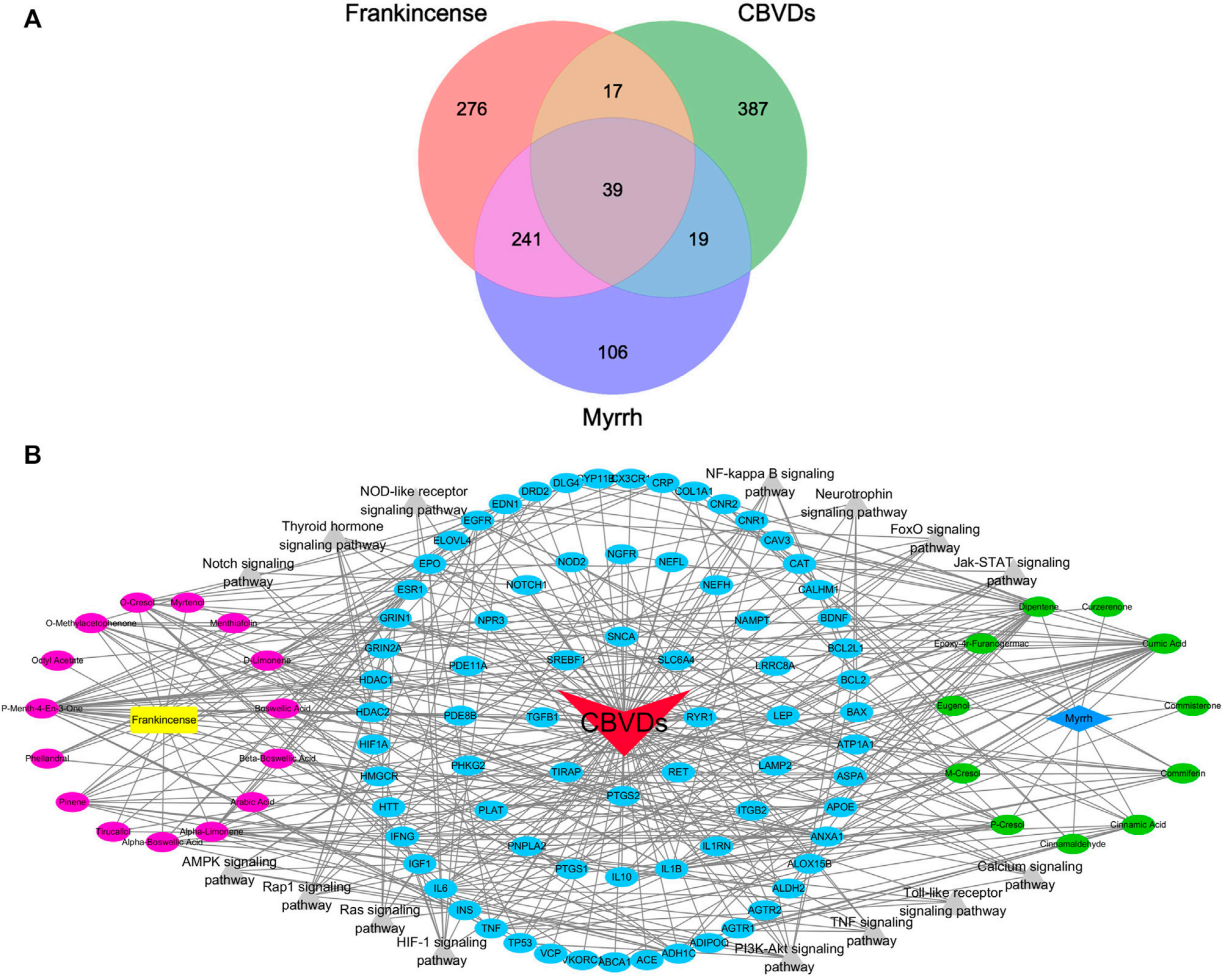
The component-target-pathway network of drugs and diseases. **(A)** Wayne figure: 75 targets that are common to Frankincense, Myrrh and CBVDs. **(B)** Visualized network diagram of component-target-pathway. Note: red triangle: CBVDs; yellow rectangle: Frankincense; blue diamond: Myrrh; red ellipse: components in Frankincense; green ellipse: components in Myrrh; blue ellipse: common targets; gray triangle: signaling pathways.

In the BATMAN-TCM database (score cutoff = 20, adjusted *p*-value = 0.05), we identified a total of 15 active compounds and 573 target genes related to Frankincense, and 11 active compounds, and 405 target genes related to Myrrh. Using the OMIM, NCBI, CTD, TTD, GeneCards, and DrugBank databases, we identified 462 targets related to CBVDs. The Wayne figure was drawn using Venny2.1, and a total of 75 targets were obtained (Figure 2A). A visual network diagram with 119 nodes (including 26 biologically active components, 15 signaling pathways and 75 targets) as well as 414 edges was established in Cytoscape (Figure 2B).

### Protein-Protein-Interaction Network of Common Targets

**FIGURE 3:**
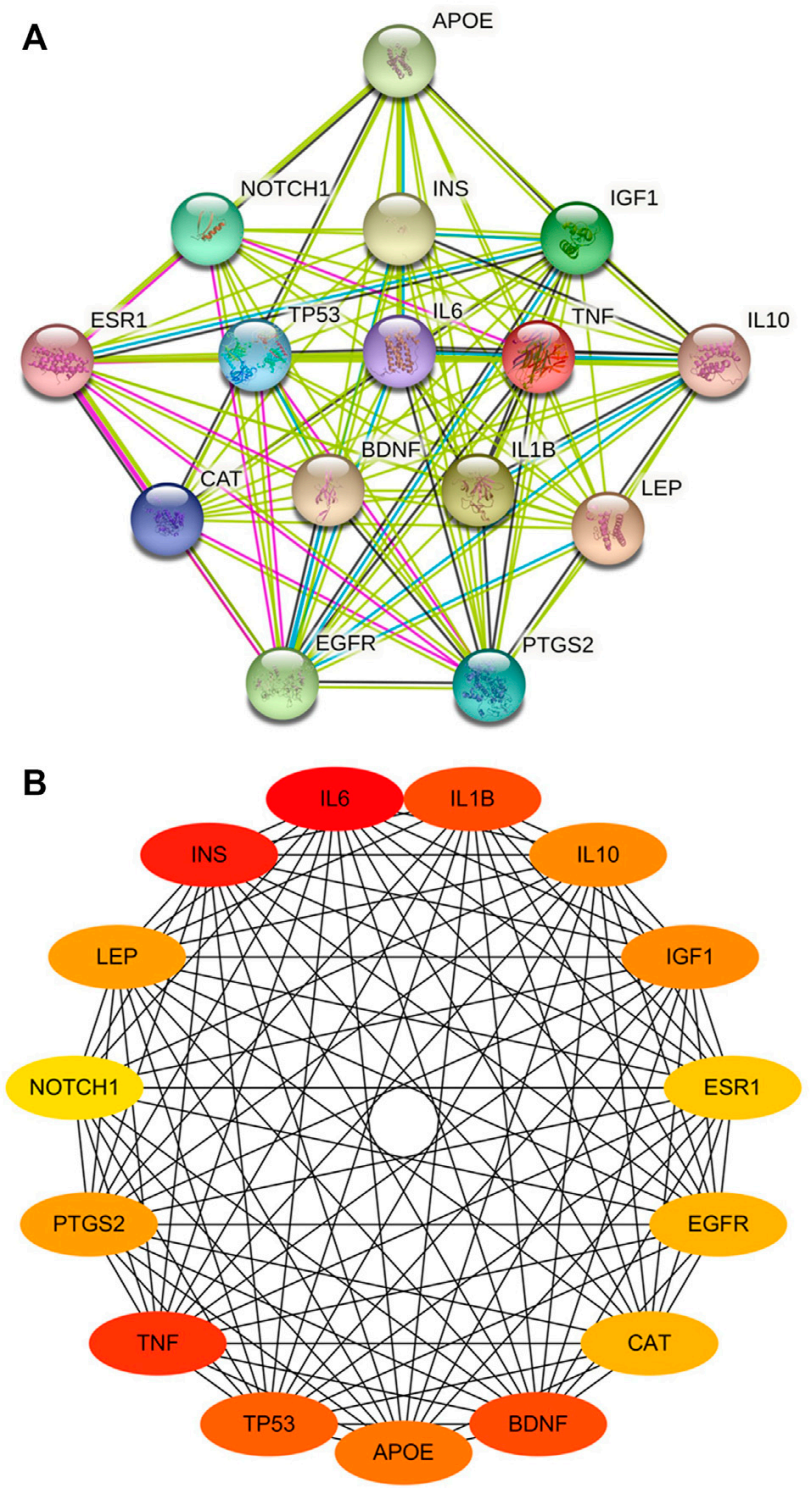
The PPI network of key targets. **(A)** Top 15 genes in degree, filled nodes represent some 3D structure is known or predicted. **(B)** Visualized network diagram of core targets. The more related proteins in the network are described in darker colors.

Overall, 75 common targets were uploaded to the STRING database to obtain the PPI network. A total score greater than 0.4, and “Homo sapiens” were selected for filtering the targets. The top 15 genes were identified as key genes of the PPI network (Figure 3A). Cytoscape was employed to build a PPI network with 15 nodes and 102 edges. In this network, more relevant proteins are described in darker colors (Figure 3B). From the PPI analysis, it can be seen that *IL6, TNF, IL1β, BDNF* and *PTGS2* are highly correlated with CBVDs.

### GO and KEGG Enrichment Analysis

**FIGURE 4:**
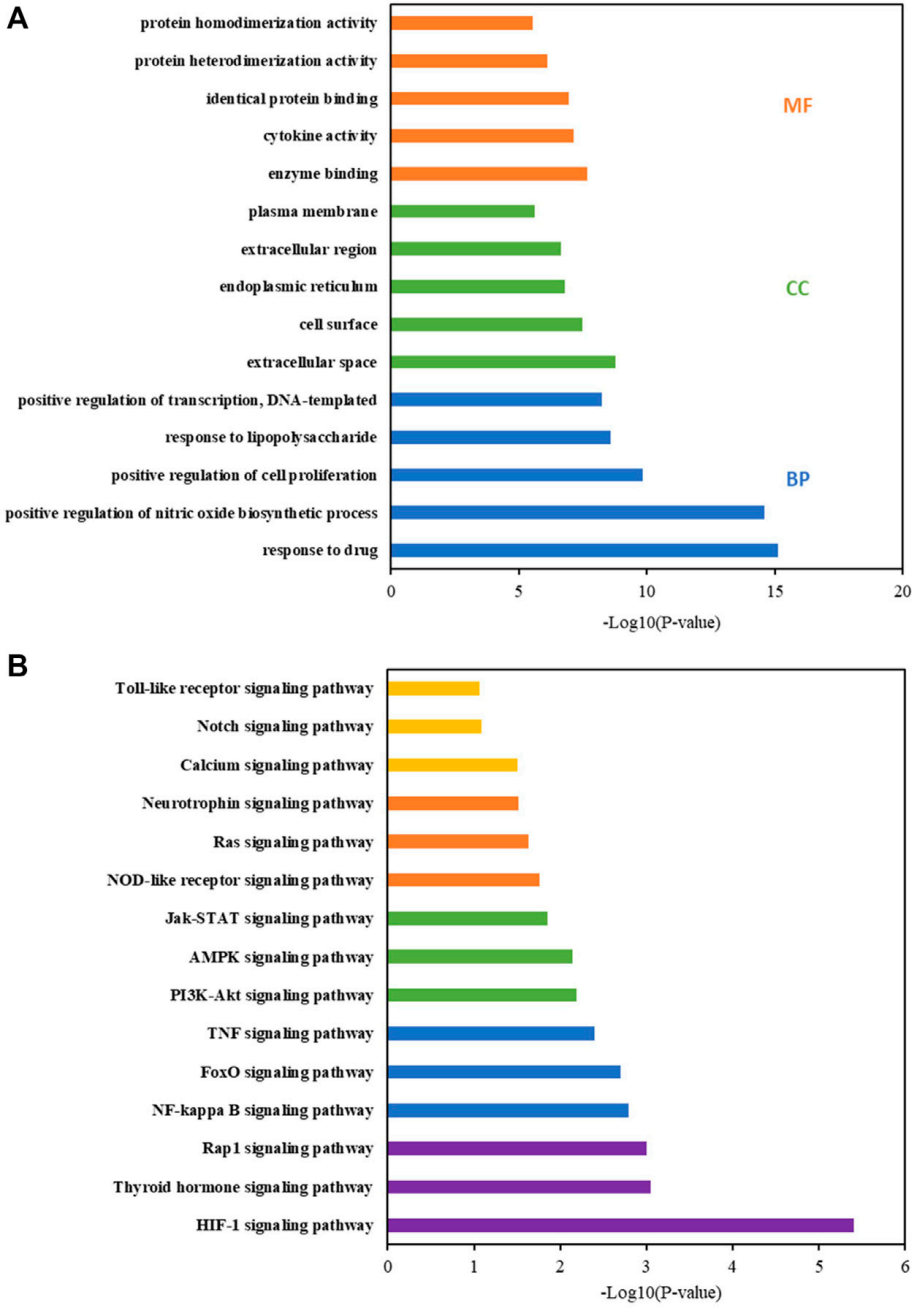
GO and KEGG pathway enrichment analyses by DAVID database. **(A)** GO term analysis: blue, green and orange bars indicate biological process (BP), cellular component (CC), and molecular function (MF), respectively. **(B)** KEGG pathway enrichment.

The DAVID database was used for the enrichment analysis of the GO and KEGG pathways. The results of GO enrichment analysis were classified into biological processes (BPs), cellular components (CCs), and molecular functions (MFs). The 320 BPs, 41 CCs, and 42 MFs enriched against these targets were statistically significant (*p* < 0.05). The first five enrichment conditions for BP, CC, and MF are shown in Figure 4A. In this study, the core KEGG pathways were screened using the DAVID database, and the first 15 signaling pathways were presented in the form of histograms, as shown in Figure 4B. According to the analysis results, the neurotrophin signaling pathway and inflammation-related (NF-kappa B, TNF, Jak-STAT, and Toll-like receptor) signaling pathways are closely related to CBVDs.

### Synergistic Amelioration by Frankincense-Myrrh of Brain Injury Induced by Middle Cerebral Artery Occlusion in Rats

**FIGURE 5:**
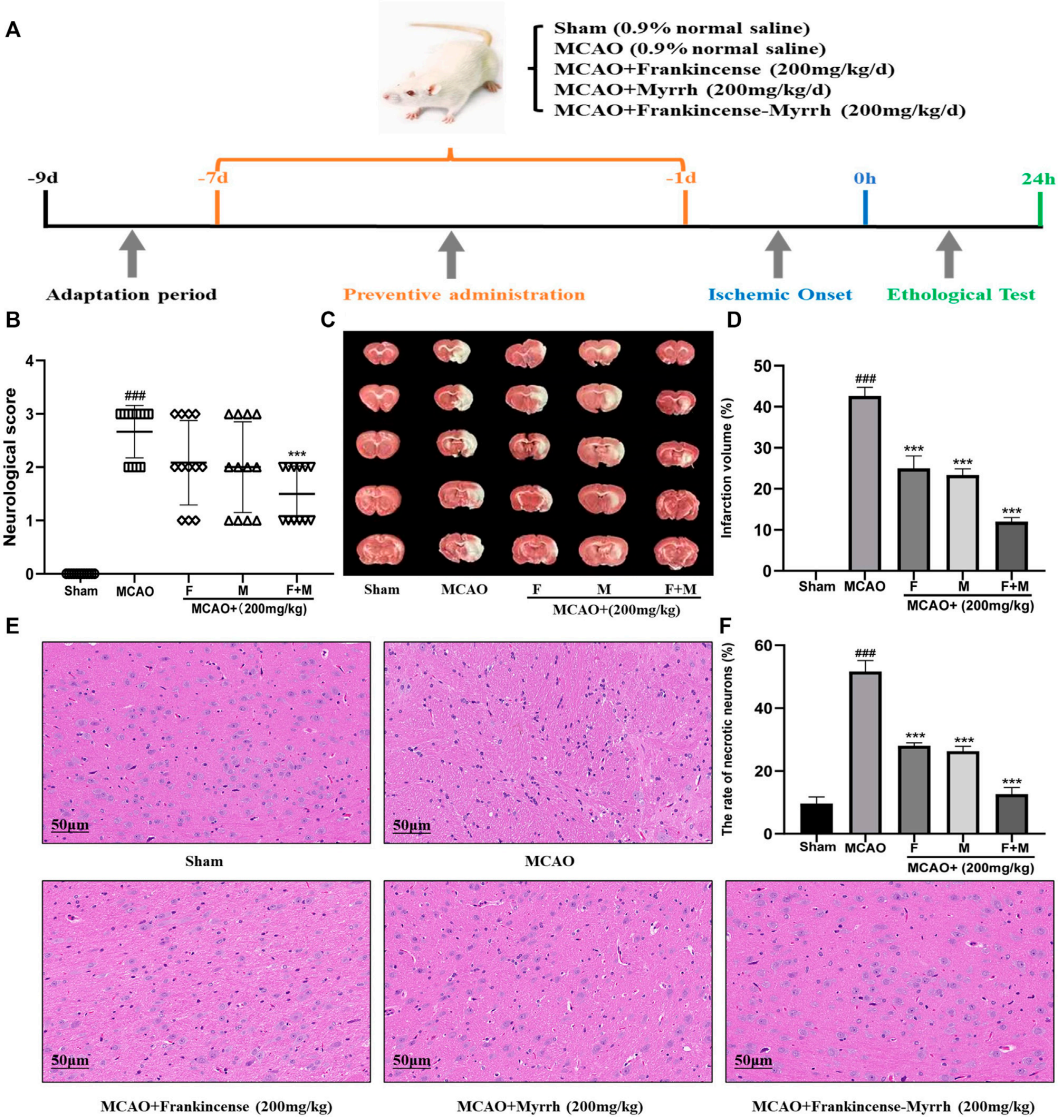
Frankincense-Myrrh synergistic ameliorates MCAO-induced brain injury. The rats were randomly divided into five groups (*n* = 12): Sham, MCAO, MCAO + Frankincense (F), MCAO + Myrrh (M) and MCAO + Frankincense-Myrrh (F+M). **(A)** Time-line graph of animal experiments. **(B)** Neurologic score of the rats. **(C)** TTC staining of the brain slices **(D)** Quantitative analysis of the infarct volume. **(E)** H&E staining of brain tissues (Scale bar = 50 μm). **(F)** Statistical analysis results of the rate of necrotic neurons in each group. All statistical data are presented as mean ± SD. ^###^ *p* < 0.001, compared with the sham group; **p < 0.01 or ***p < 0.001, compared with the MCAO group.

Neurologic deficits were scored before the rats were sacrificed. Results showed that MCAO induced severe functional impairment, with a significant decrease in scores in the group treated with frankincense + myrrh (Figure 5B). TTC staining showed no area of damage in the contralateral cerebral hemisphere in the sham and model (MCAO) groups. In contrast, the ipsilateral cerebral hemispheres in the operated group showed extensive lesions (Figure 5C). Quantitative analysis of infarct volumes showed that Frankincense and Myrrh treatments significantly reduced the extent of cerebral infarction (Figure 5D). H&E staining showed histopathological changes. While cells in the sham group were neatly arranged without any morphological changes, obvious symptoms such as disordered neuron arrangement, pyknotic nucleus, and neuronal loss appeared after ischemia; Frankincense and Myrrh treatment ameliorated these histopathological damages (Figures 5E,F). All data showed that the effects of combinations of these two natural products were greater than those of one product alone. The above findings reveal that the combination of Frankincense and Myrrh could reduce brain damage, enhance neuroprotection effects, and contribute to brain function restoration after cerebral ischemia. Thus, Frankincense and Myrrh have a synergistic protective effect against CBVDs.

### Inhibition of Microglial Expression and Promotion of Neuronal Expression by Frankincense-Myrrh

**FIGURE 6:**
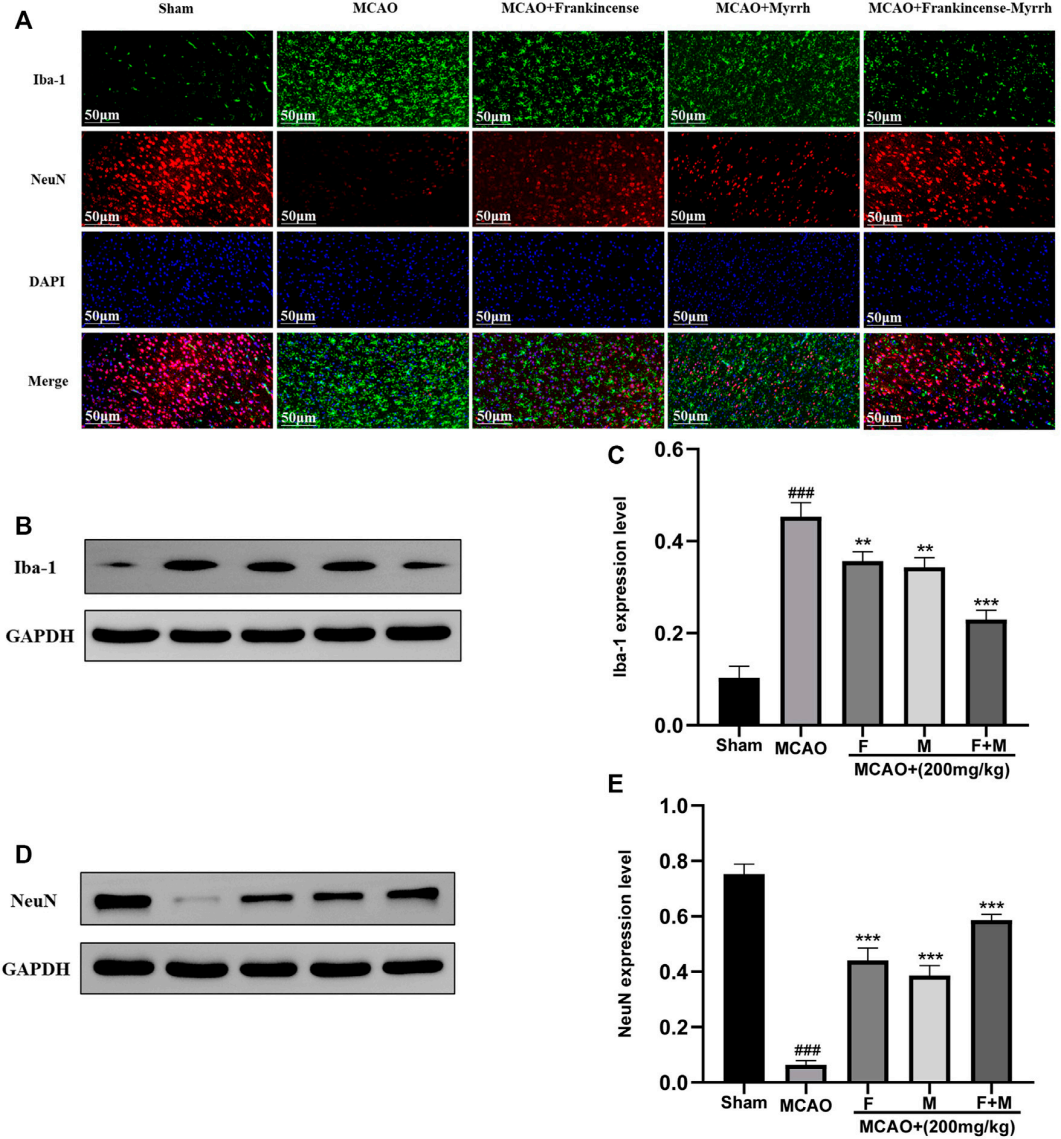
Frankincense-Myrrh inhibiting the expression of microglia marker Iba-1, While promotes the expression of neurons marker NeuN. **(A)** Immunofluorescence staining for Iba-1 and NeuN. **(B)** Quantitative results of the protein Iba-1 expression. **(C)** Statistical analysis results of detection protein Iba-1. **(D)** Quantitative results of the protein NeuN expression. **(E)** Statistical analysis results of detection protein NeuN. All statistical data are presented as mean ± SD. ^###^ *p* < 0.001, compared with the sham group; **p < 0.01 or ***p < 0.001, compared with the MCAO group.

To verify the expression of microglia and neurons in CBVDs, immunofluorescence and western blot were used to detect the expression of the marker proteins Iba-1 and NeuN. Immunofluorescence results showed that Frankincense and Myrrh inhibited the expression of microglia and promoted neuronal expression (Figure 6A). Western blot results showed that the microglial marker Iba-1 was rarely expressed in the sham group, while the expression increased significantly after ischemia; however, Frankincense and Myrrh significantly inhibited the expression of microglia (Figures 6B,C). The neuronal marker NeuN was highly expressed in normal brain tissue, and decreased with cerebral injury. The reduced expression was partially restored after Frankincense and myrrh treatment (Figures 6D,E). All data showed that the effects of combinations of the two natural products were greater than those of the individual products.

### Regulation of *BDNF, COX-2, IL6, IL1β and TNF-α* Expressions by Frankincense-Myrrh in Rats Induced With Middle Cerebral Artery Occlusion

**FIGURE 7:**
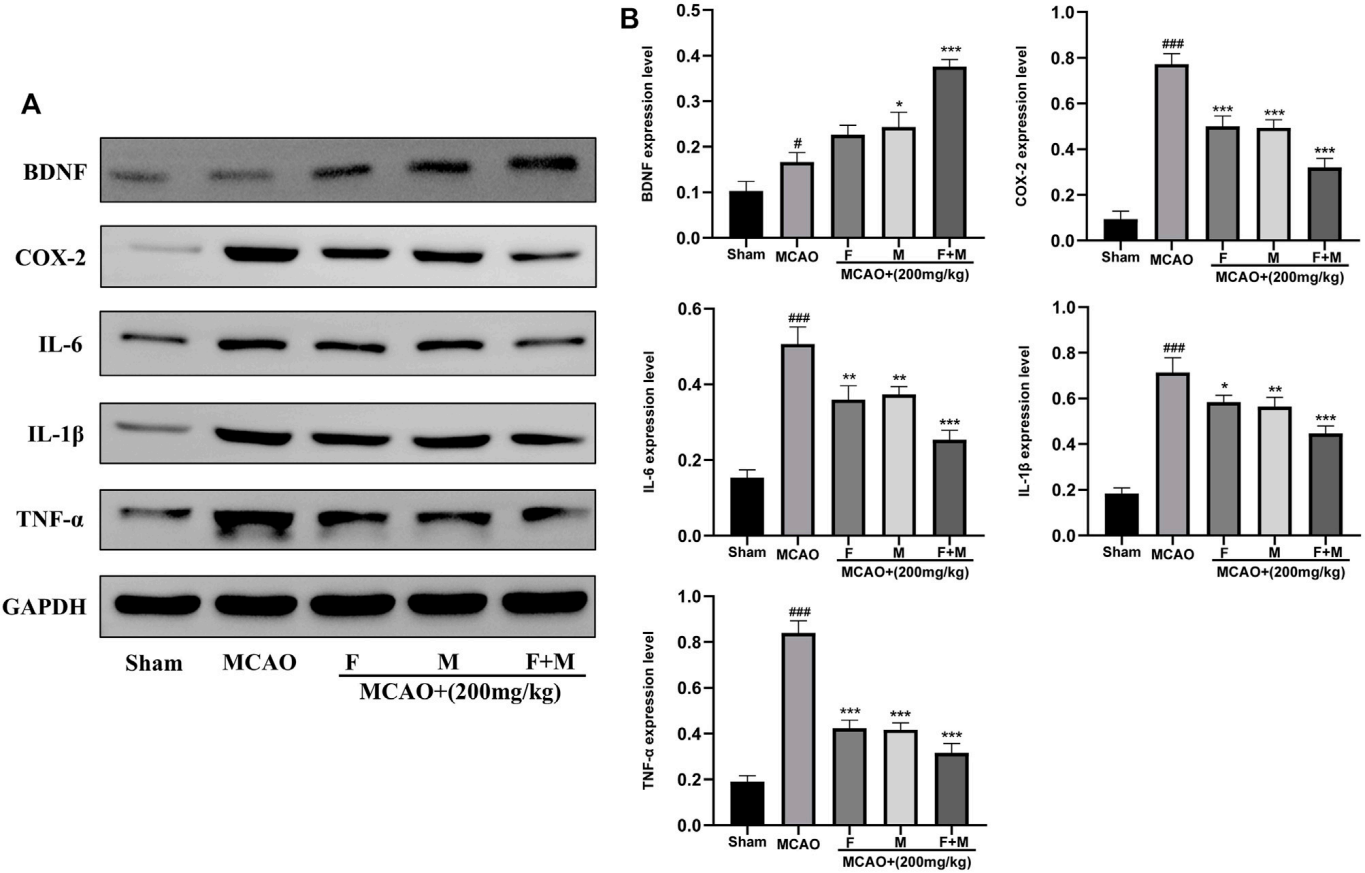
Relative expression of BDNF, COX-2, IL-6, IL-1β and TNF-α in the cerebral cortex of the Sham, MCAO, MCAO + Frankincense, MCAO + Myrrh and MCAO + Frankincense-Myrrh pretreatment groups were detected by using Western blot analysis. **(A)** Representative bands exhibited the relative expression of BDNF, COX-2, IL-6, IL-1β and TNF-α; **(B)** Statistical analysis results of detected proteins in each group. Data were presented as mean ± SD. ^#^ *p* < 0.05, ^###^ *p* < 0.001, compared with the sham group; *p < 0.05, **p < 0.01 or ***p < 0.001, compared with the MCAO group.

To verify the reliability of potential targets determined by bioinformatic analysis, western blot was used to detect the expression of neurotrophic factors and pro-inflammatory factors. The results showed that the expression of the neurotrophic factor, BDNF protein, was increased slightly in the MCAO group, and markedly increased in the Frankincense-Myrrh group. Moreover, the pro-inflammatory factors *COX-2, IL-6, IL-1β, and TNF-α* were rarely expressed in the sham group, while their expression was remarkably elevated after ischemia. However, Frankincense and Myrrh significantly inhibited the expression of *COX-2, IL-6, IL-1β, and TNF-α*. The effects were more pronounced for the combination of Frankincense and Myrrh (Figures 7A,B). These results indicate that Frankincense and Myrrh can synergistically increase neurotrophic factors in rats afflicted with ischemic stroke, by inhibiting the expression of inflammatory factors.

### Regulation by Frankincense-Myrrh of the Expression of *BDNF, COX-2, IL6, IL1β* and *TNF-α* in Brain Microvascular Endothelial Cells Subjected to Oxygen-Glucose Deprivation

**FIGURE 8:**
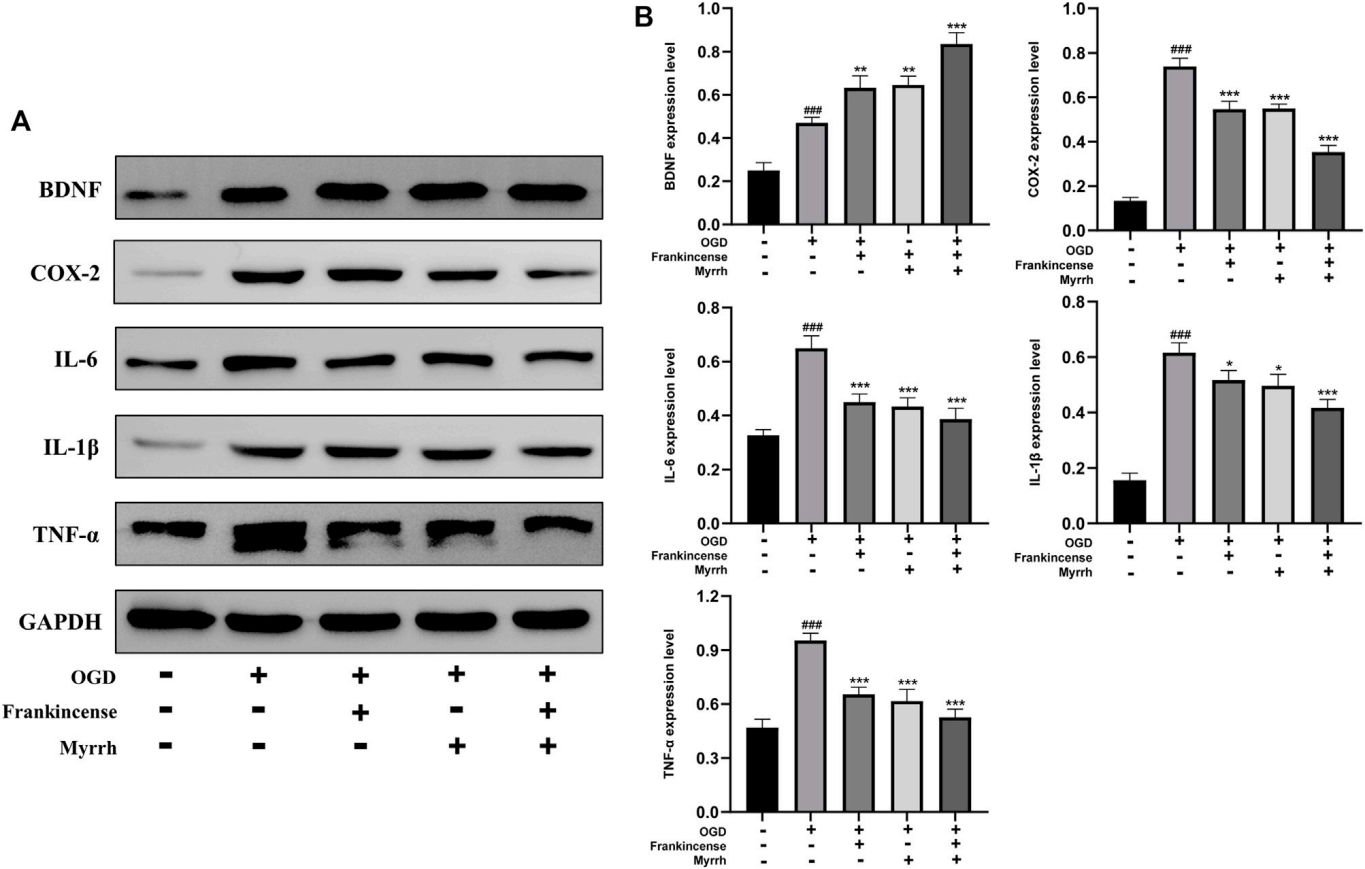
Relative expression of BDNF, COX-2, IL-6, IL-1β and TNF-α in the BMECs of the Control, OGD, OGD + Frankincense, OGD + Myrrh and OGD + Frankincense-Myrrh pretreatment groups were detected by using Western blot analysis. **(A)** Representative bands exhibited the relative expression of BDNF, COX-2, IL-6, IL-1β and TNF-α; **(B)** Statistical analysis results of detected proteins in each group. Data were presented as mean ± SD. ^###^ *p* < 0.001, compared with the control group; *p < 0.05, **p < 0.01 or ***p < 0.001, compared with the OGD group.

To explore whether Frankincense and Myrrh produce the same effect *in vitro*, BMECs in the rat brain were subjected to OGD. Western blot was used to detect the expression of neurotrophic and pro-inflammatory factors. The results showed that after BMECs were subjected to OGD, Frankincense and Myrrh promoted the expression of BDNF, while inhibiting the expression of *COX-2, IL6, IL1β and TNF-α* (Figures 8A,B). All data showed that the effects of combinations of the two products were greater than those of the individual products. *In vitro* experiments indicated that Frankincense-Myrrh treatment significantly increased the levels of neurotrophic factors in BMECs subjected to OGD, while inhibiting pro-inflammatory factors. The synergistic effect may occur at the level of signal transduction, which is consistent with the results of the *in vivo* experiments.

## Discussion

According to the “Compendium of Materia Medica” that includes records on TCM, Frankincense-Myrrh is a classic drug pair, and both drugs indicate clear clinical effects and safety in the treatment of CBVDs (Liu et al., 2018; Baram et al., 2019). However, though the combination of Frankincense and Myrrh has a synergistic effect, the biologically active ingredients, and the multi-target mechanism that produces those effects have not been clearly elucidated. In this study, 75 common targets were selected by matching the potential targets of Frankincense-Myrrh and the target genes related to CBVDs. Fifteen core targets were identified by analyzing the PPI network. GO functional and KEGG pathway enrichment analyses were performed to better understand the reciprocity of these common targets. The enriched results suggested that the synergistic mechanism of Frankincense-Myrrh on CBVDs were predominately involved in neurotrophin signaling pathway and inflammation-related (NF-kappa B, TNF, Jak-STAT, and Toll-like receptor) signaling pathways. Finally, by establishing MCAO in rats and subjecting BMECs in rats to OGD, various experimental evaluations (including neurological scale scores, TTC staining, H&E staining, immunofluorescence and western blot) were carried out to verify the accuracy of the prediction arrived at through systems pharmacology and *in vivo* and *in vitro* experiments.

Interestingly, the combination of Frankincense and Myrrh in the treatment of CBVDs achieved better results than monotherapy involving only one of them. This suggests that it has a synergistic protective effect against CBVDs. Research on the material basis and synergistic mechanism of the Frankincense-Myrrh compound is still in its infancy. Studies have found that the dissolution of pentacyclic triterpenoids increased significantly after the combination of Frankincense and Myrrh, while the dissolution of sesquiterpenoids decreased (Shen and Lou, 2008; Su et al., 2012). This indicates that changes in the chemical composition of Frankincense and Myrrh may be related to the physical changes and chemical reactions that occur during the merging process, such as solubilization, oxidation, reduction, and hydrolysis (Marongiu et al., 2005; Cao et al., 2019). Whether the change of each component is the material basis for the synergistic effect of the Frankincense–Myrrh compound needs to be explored further.

Brain-derived neurotrophic factor (BDNF) is a member of the neurotrophic factor family and plays an important role in the development of the nervous system and the formation of synapses (Ramos-Cejudo et al., 2015; Monnier et al., 2017). Han et al. (2015) observed that BDNF plays an important role in the differentiation of bone marrow mesenchymal stem cells into neuronal cells. Grade et al. (2013) confirmed that BDNF can promote the migration of neuronal precursor cells to the ischemic area and promote nerve regeneration. Therefore, increased BDNF expression after cerebral ischemia-reperfusion injury induces neuronal precursor cells to migrate and differentiate into mature nerve cells, stimulate nerve regeneration, promote the repair of damaged nerves, and reduce cell apoptosis. These factors may be an effective way to treat CBVD. Cerebral ischemia-reperfusion injury caused by an inflammatory response is a complex cascading pathological process that not only affects the blood supply to the injured area, but also directly destroys the tissue structure (Ramiro et al., 2018; Walas et al., 2019). PTGS2, also known as cyclooxygenase-2 (COX-2), is the major isoenzyme responsible for inflammatory prostaglandin production, and its inhibition may help ameliorate infarct expansion after CBVDs (Beaudin et al., 2014). Microglia play an important role in this process. Microglia can be activated by neurotoxic substances such as cell fragments, reactive oxygen species, and NO produced by neuronal necrosis caused by cerebral ischemia; this can then produce a large number of inflammatory factors such as interleukin 6 (IL6), interleukin 1β (IL1β), and tumor necrosis factor alpha (TNF-α), causing local brain tissue inflammation, infiltration, and damage (Yu et al., 2016; Koizumi et al., 2019; Aliena-Valero et al., 2021). Excessive activation of microglia releases a large amount of pro-inflammatory factors, which is a direct factor that aggravates cerebral ischemia-reperfusion injury (Klement et al., 2018; Naveed et al., 2019). In cerebral ischemia, abnormal activation of inflammatory pathways and a significant increase in the secretion of pro-inflammatory cytokines occur (Zhao et al., 2017; Chen et al., 2019). Therefore, inhibiting the release of pro-inflammatory factors in microglia may be the main mechanism for alleviating cerebral ischemia-reperfusion injury.

Our research shows that the intervention of Frankincense-Myrrh in the rat model of cerebral ischemia-reperfusion injury can significantly reduce the extent of ischemic cortical infarction, promote the expression of BDNF and NeuN, and inhibit the expression of *Iba-1, COX-2, IL-6, IL-1β*, and *TNF-α.* It has been suggested that increased expression of neurotrophic factors after cerebral ischemia-reperfusion injury, inhibition of microglial activation and neuronal cell apoptosis, and reduction in the release of inflammatory factors are the potential mechanisms of its synergistic effect on the brain. However, the study has some limitations. The correction for multiple testing has not been applied to the GO and KEGG enrichment analysis, and false positive results may occur; the interaction between microglia and neurons is extremely complex and involves multiple signaling molecules on which greater research is required; the remarkable synergy of Frankincense and Myrrh is also worthy of further study. Therefore, more *in vitro, in vivo*, and clinical studies are needed to verify the synergy and safety of Frankincense and Myrrh in the treatment of CBVDs; they may be involved in regulating microglia and neurons, promoting the expression of neurotrophic factors, and inhibiting the expression of inflammatory cytokines. Our research thus provides a reliable basis for the clinical application of Frankincense-Myrrh in the treatment of CBVDs.

## Conclusion

In this study, a combination of systems pharmacology and experimental evaluation was used to explore the bioactive components, therapeutic targets, and pharmacological mechanisms of Frankincense-Myrrh in the treatment of CBVDs. The study results suggest that Frankincense-Myrrh has a synergistic protective effect against CBVDs. Inhibiting the expression of microglia promotes the expression of neurons; the multi-target synergistic mechanism mainly involves the regulation of neurotrophic factors and inflammatory cytokines. In conclusion, our research provides an insight into the synergistic mechanism of Frankincense-Myrrh, and provides a scientific basis for its clinical application in the treatment of CBVDs.

## Data Availability

The raw data supporting the conclusions of this article will be made available by the authors, without undue reservation.

## Footnotes

1
http://bionet.ncpsb.org/batman-tcm/.

2
https://www.uniprot.org/.

3
https://www.ncbi.nlm.nih.gov/gene/.

4
http://omim.org/.

5
http://ctdbase.org/.

6
http://bidd.nus.edu.sg/group/cjttd/.

7
https://www.genecards.org/.

8
https://www.drugbank.ca.

9
http://bioinfogp.cnb.csic.es/tools/venny/).

10
https://string-db.org.
