# Supplementary figures and images for: Combination of Systems Pharmacology and Experimental Evaluation to Explore the Mechanism of Synergistic Action of Frankincense-Myrrh in the Treatment of Cerebrovascular Diseases
Source: Front Pharmacol. 2022 Jan 10;12:796224. doi: 10.3389/fphar.2021.796224 (PMC8784887; doi:10.3389/fphar.2021.796224)

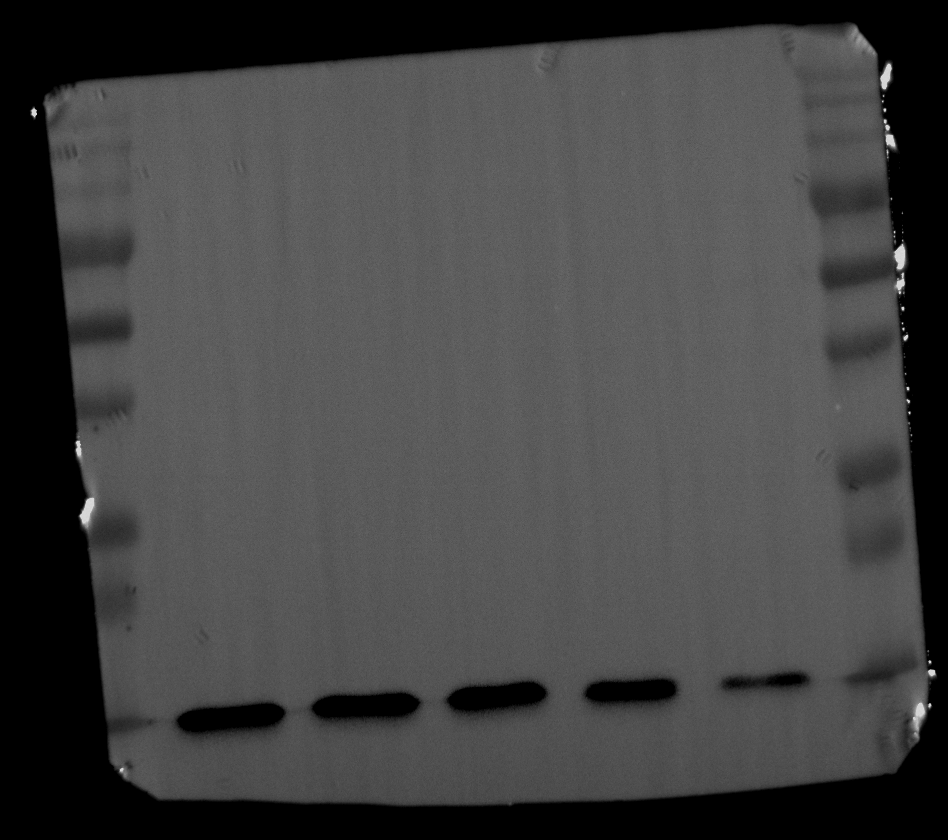

Supplement: Supplementary file 2 [file DataSheet1.zip › Western Blotting/BDNF(BMECsú⌐.tif]

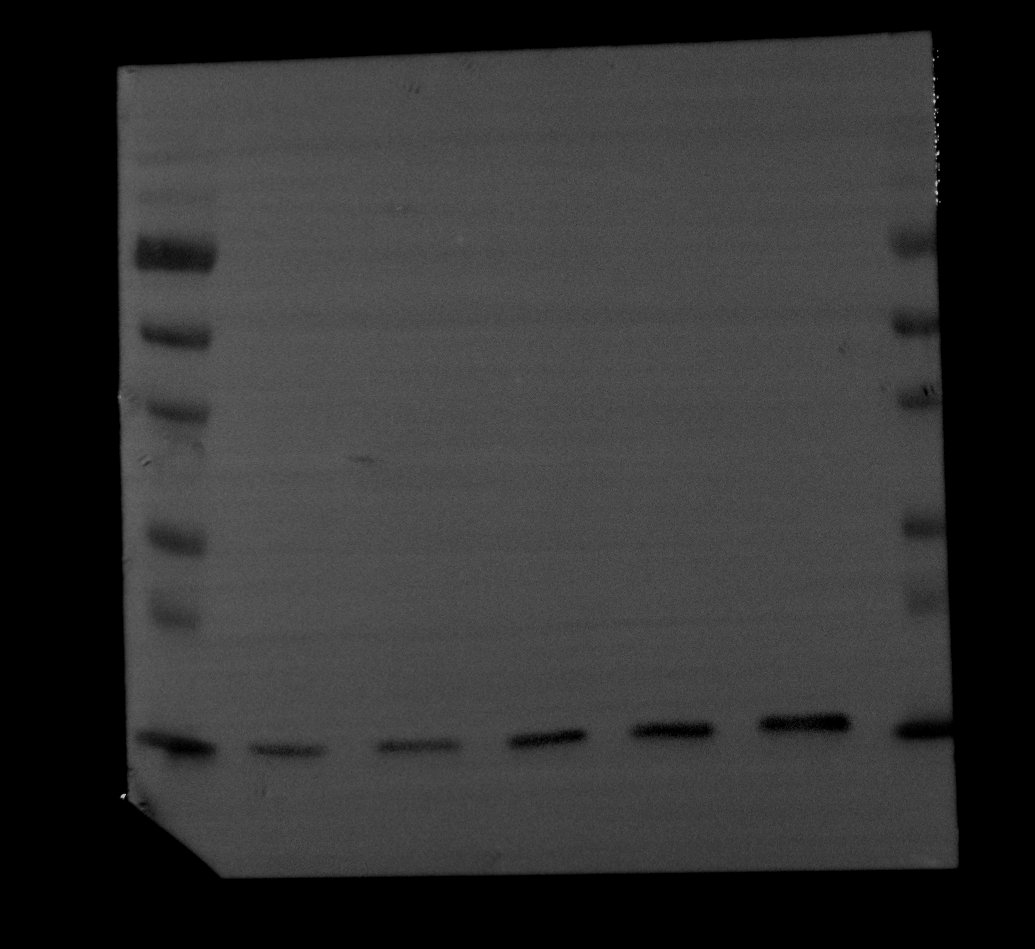

Supplement: Supplementary file 2 [file DataSheet1.zip › Western Blotting/BDNF(Ratsú⌐.tif]

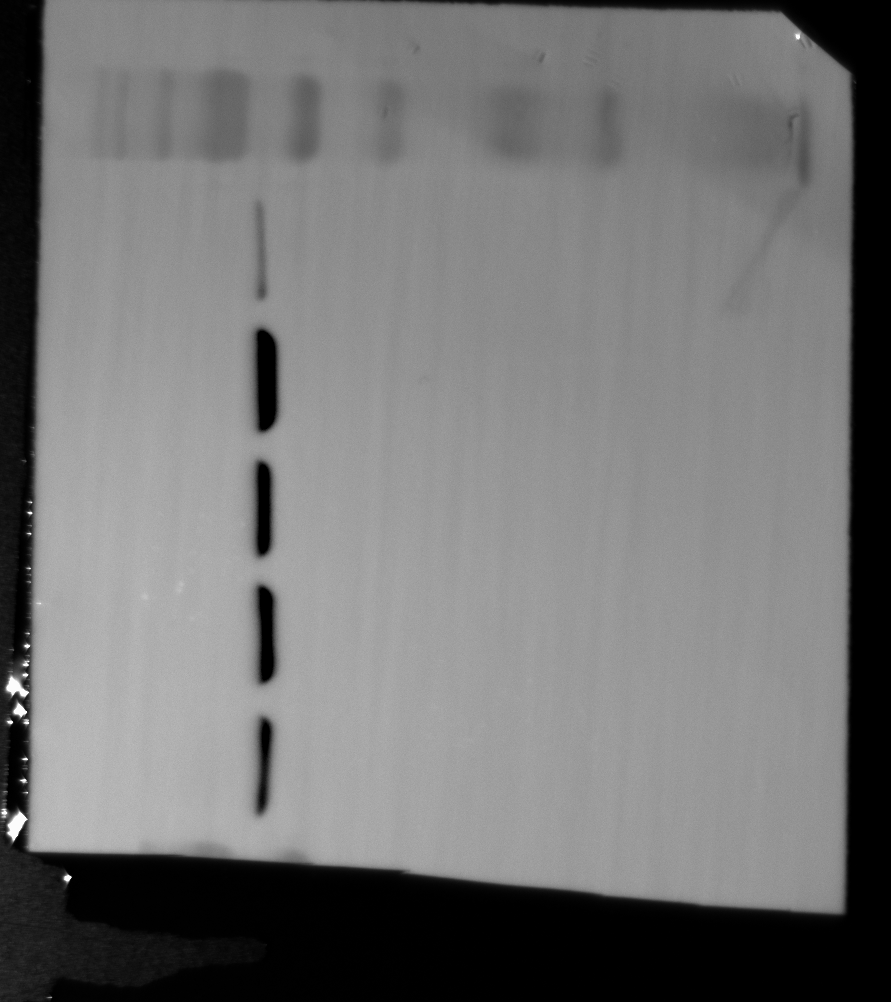

Supplement: Supplementary file 2 [file DataSheet1.zip › Western Blotting/COX-2 (Ratsú⌐.tif]

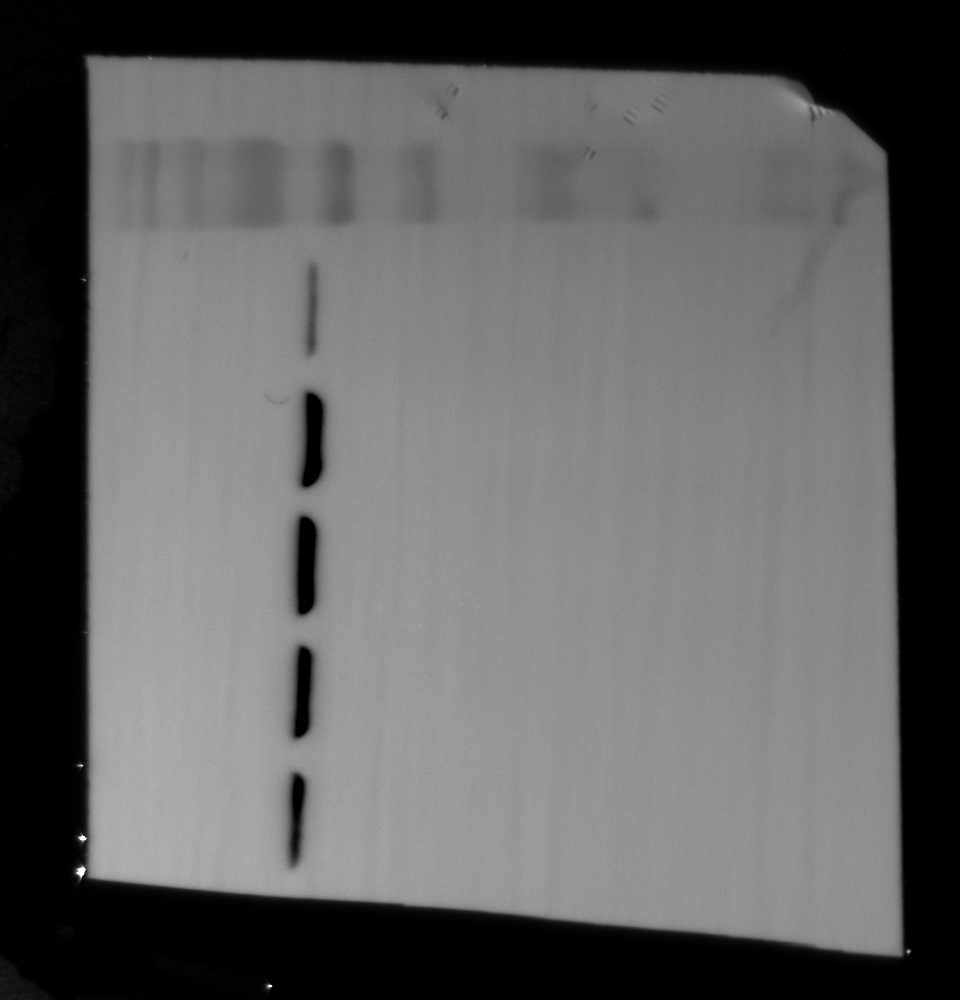

Supplement: Supplementary file 2 [file DataSheet1.zip › Western Blotting/COX-2(BMECsú⌐.tif]

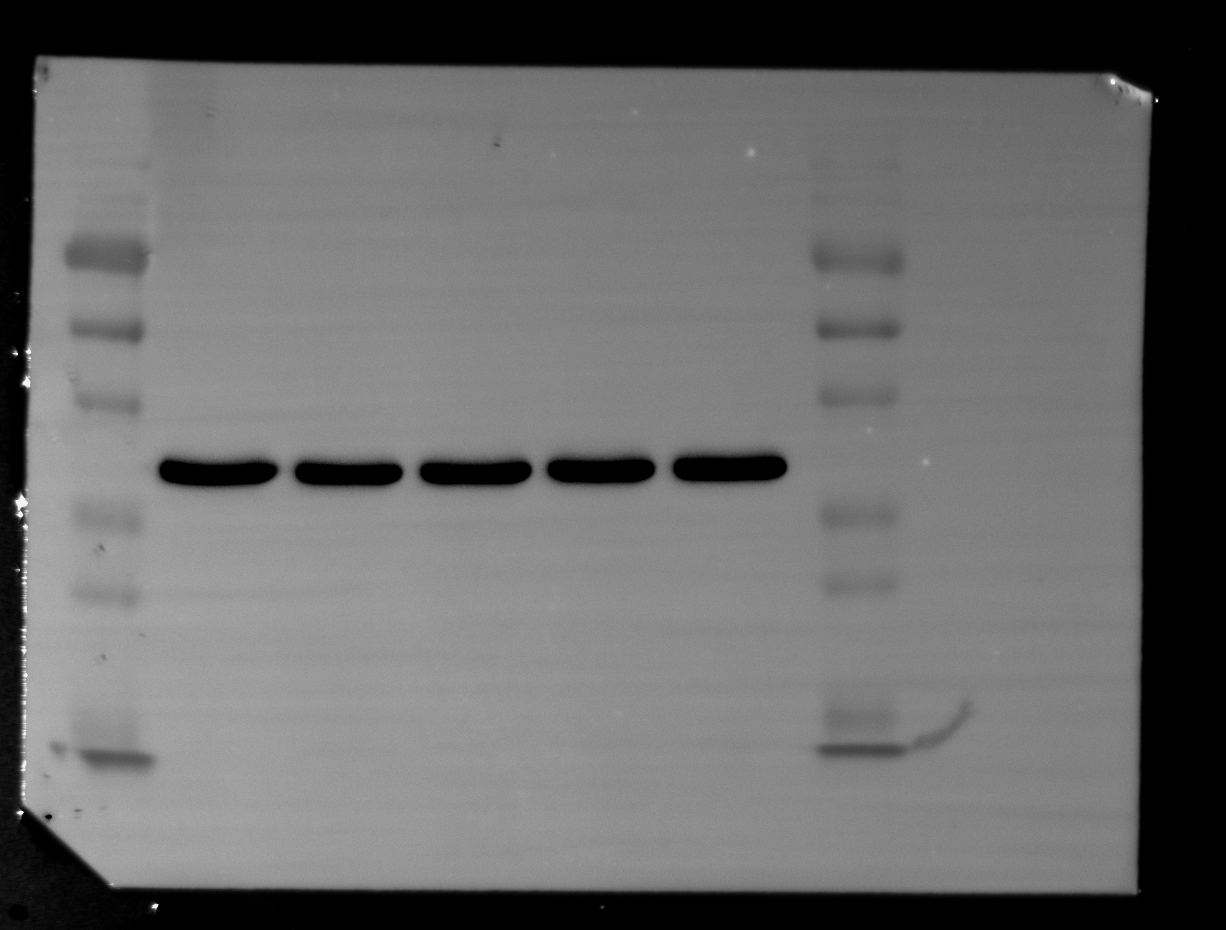

Supplement: Supplementary file 2 [file DataSheet1.zip › Western Blotting/GAPDH 1.tif]

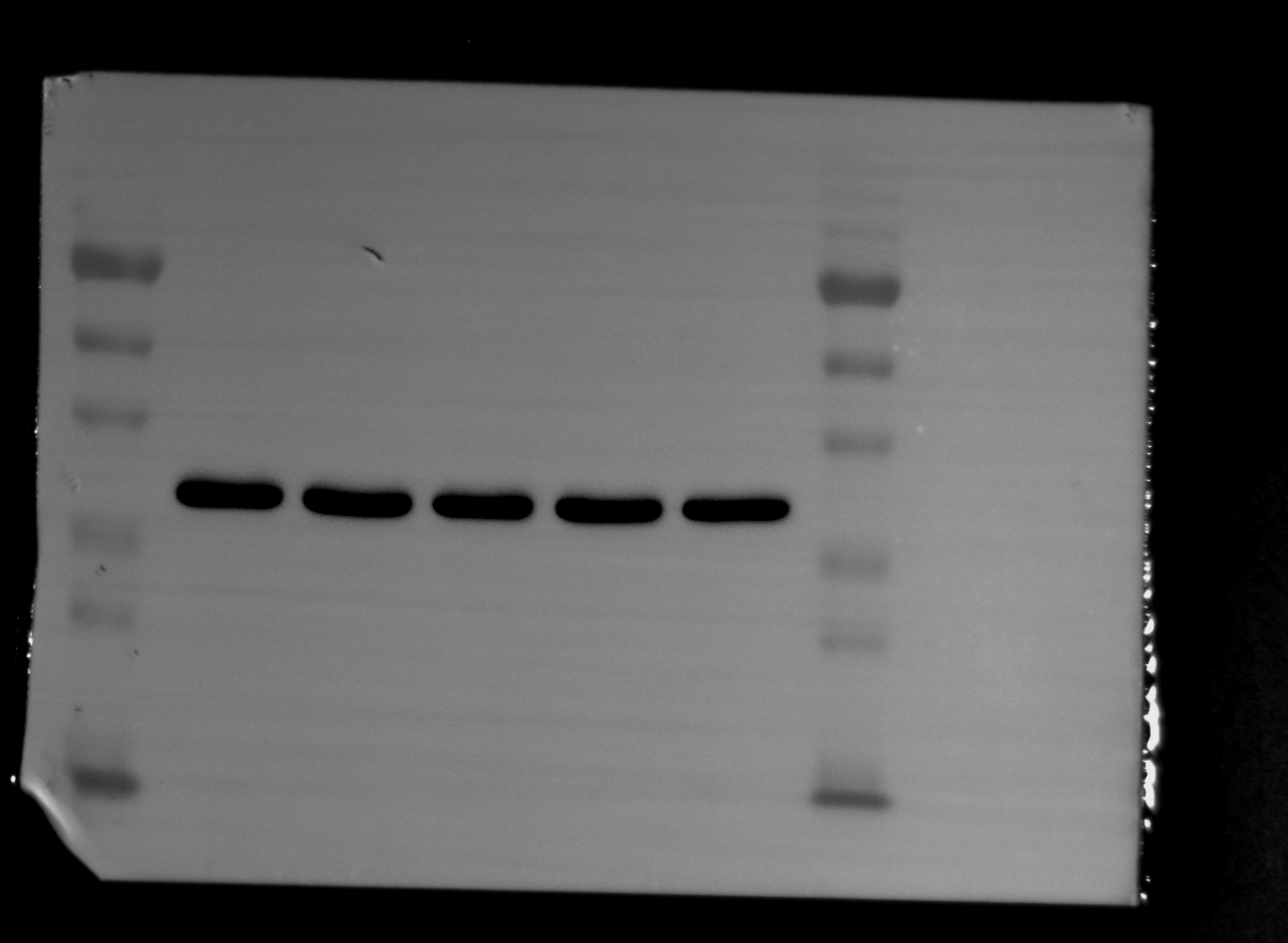

Supplement: Supplementary file 2 [file DataSheet1.zip › Western Blotting/GAPDH 2.tif]

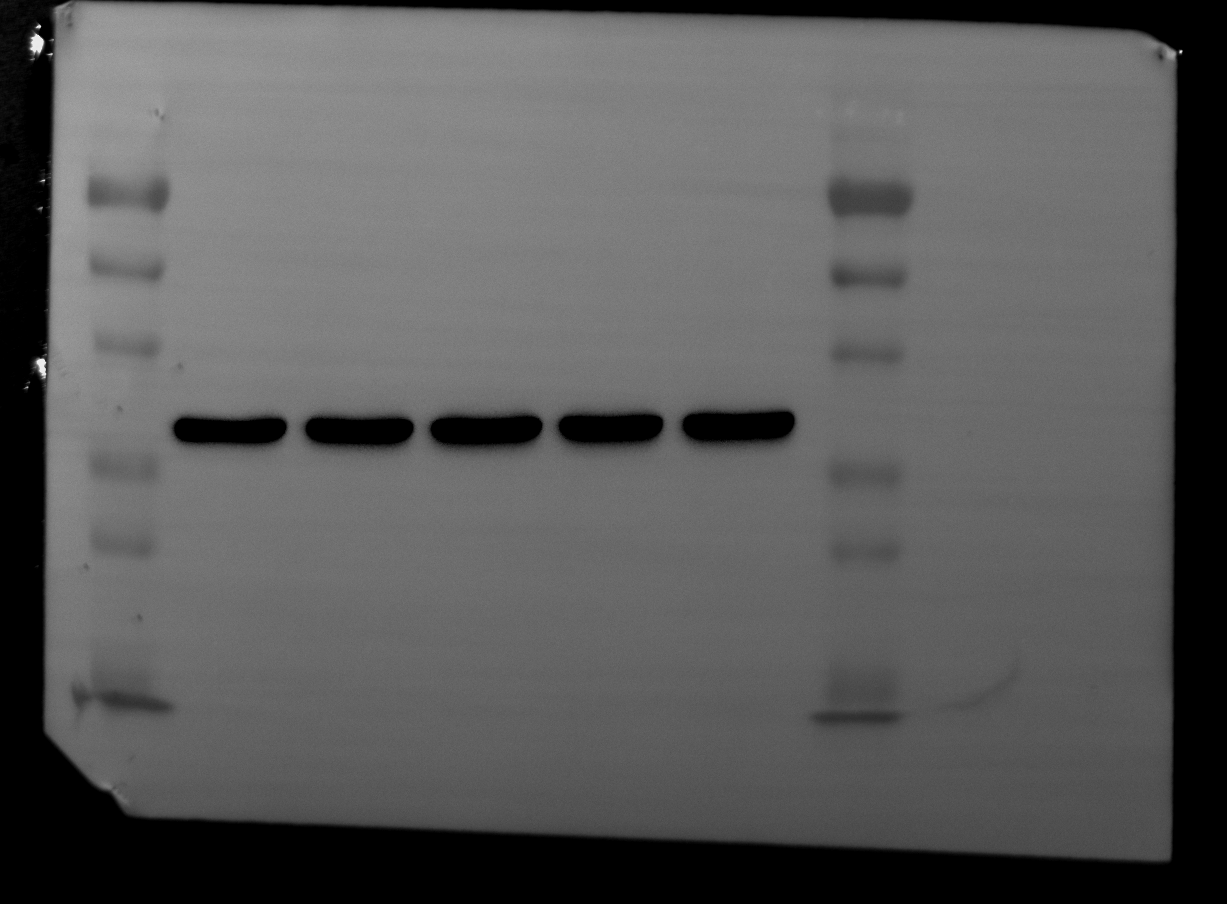

Supplement: Supplementary file 2 [file DataSheet1.zip › Western Blotting/GAPDH 3.tif]

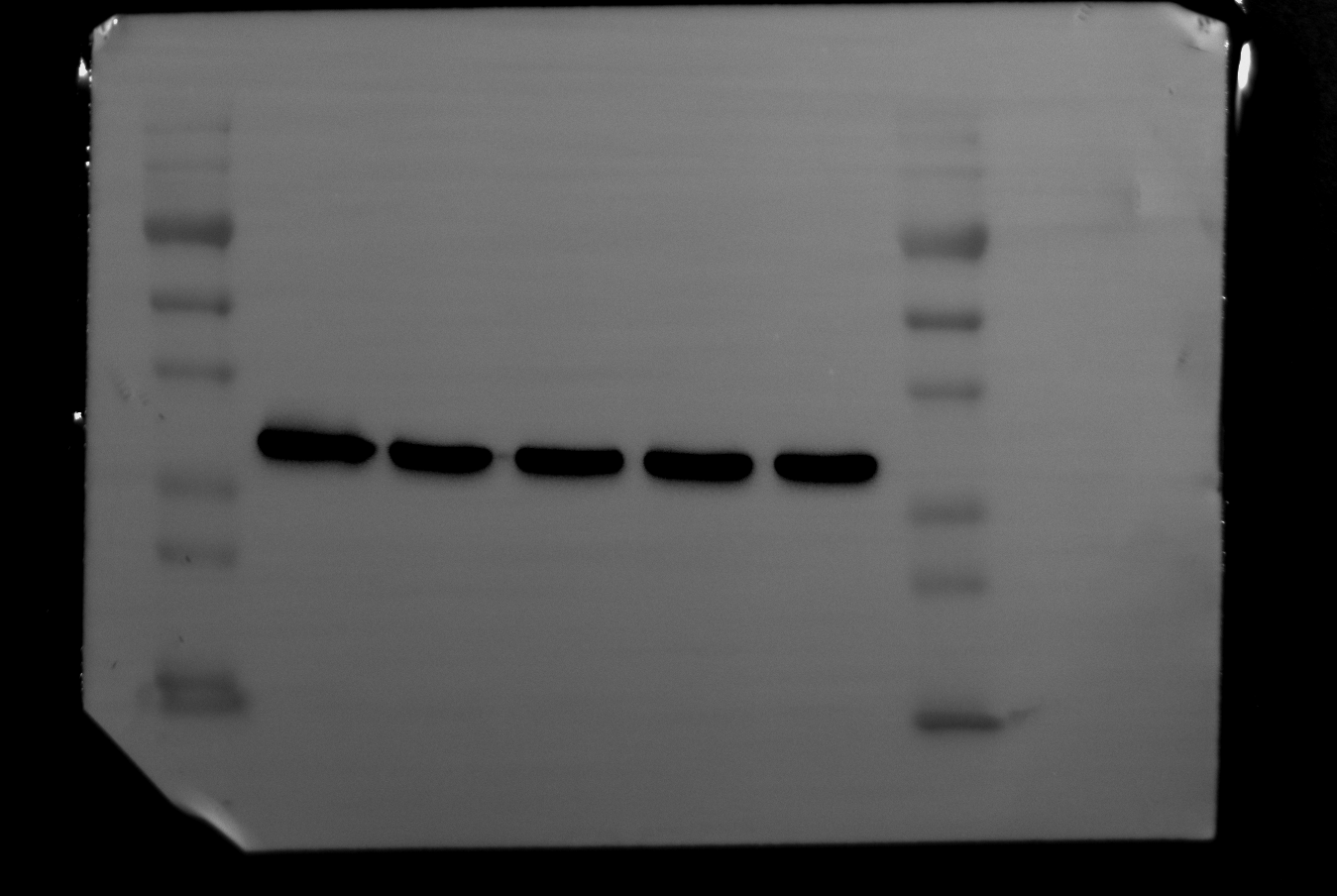

Supplement: Supplementary file 2 [file DataSheet1.zip › Western Blotting/GAPDH 4.tif]

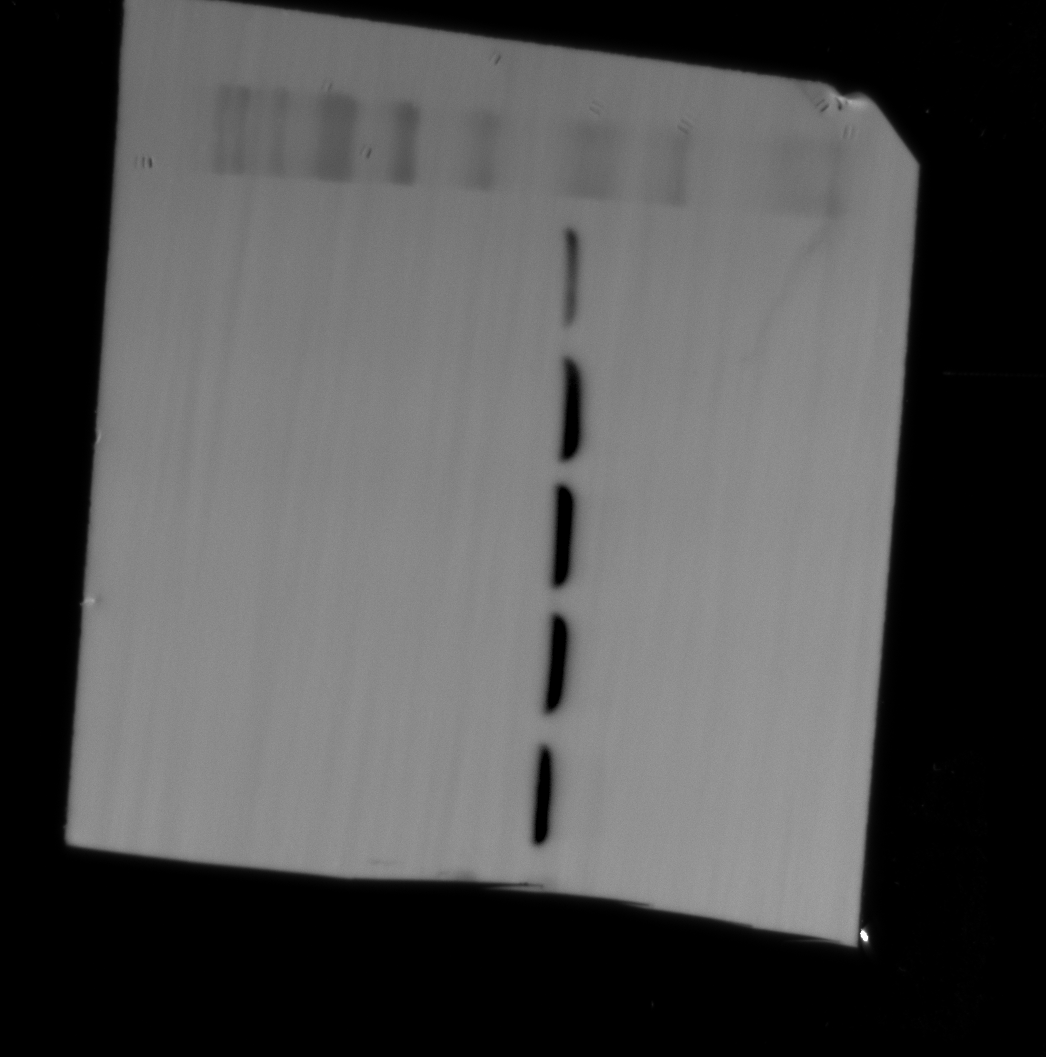

Supplement: Supplementary file 2 [file DataSheet1.zip › Western Blotting/IL-1a┬ (BMECsú⌐.tif]

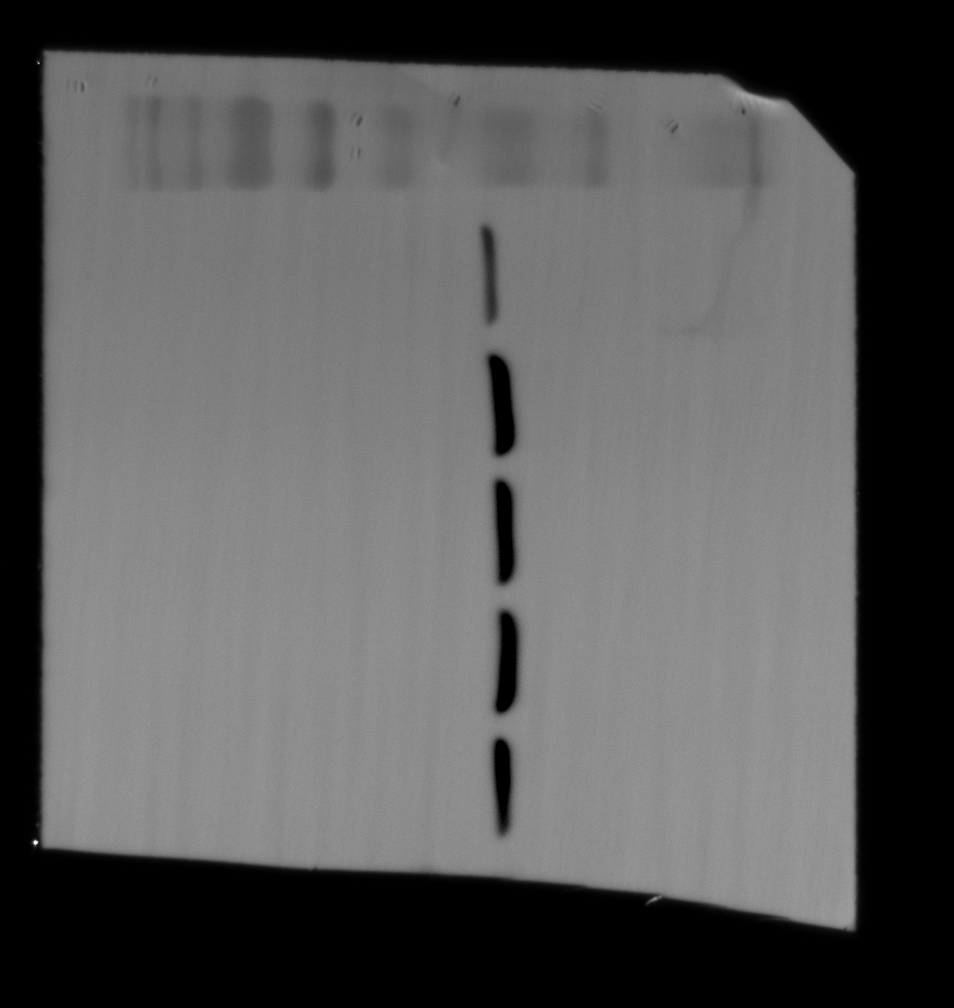

Supplement: Supplementary file 2 [file DataSheet1.zip › Western Blotting/IL-1a┬(Ratsú⌐.tif]

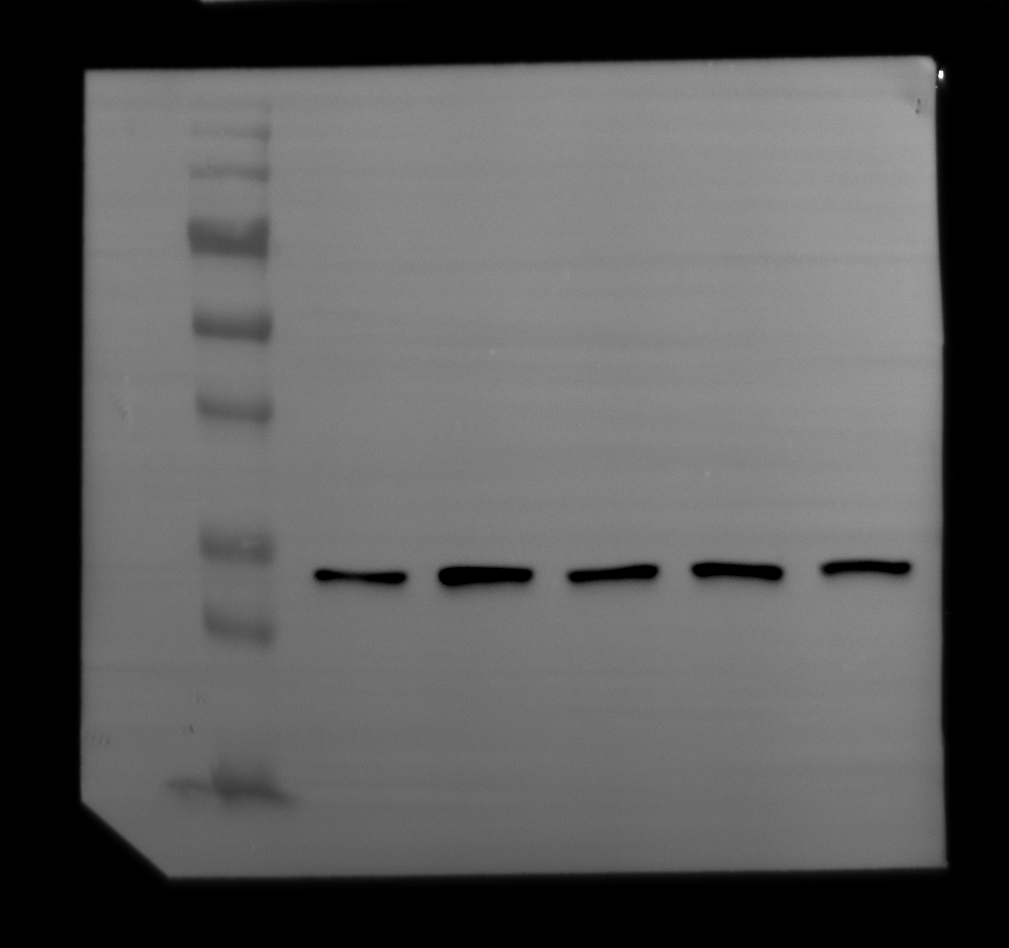

Supplement: Supplementary file 2 [file DataSheet1.zip › Western Blotting/IL-6(BMECsú⌐.tif]

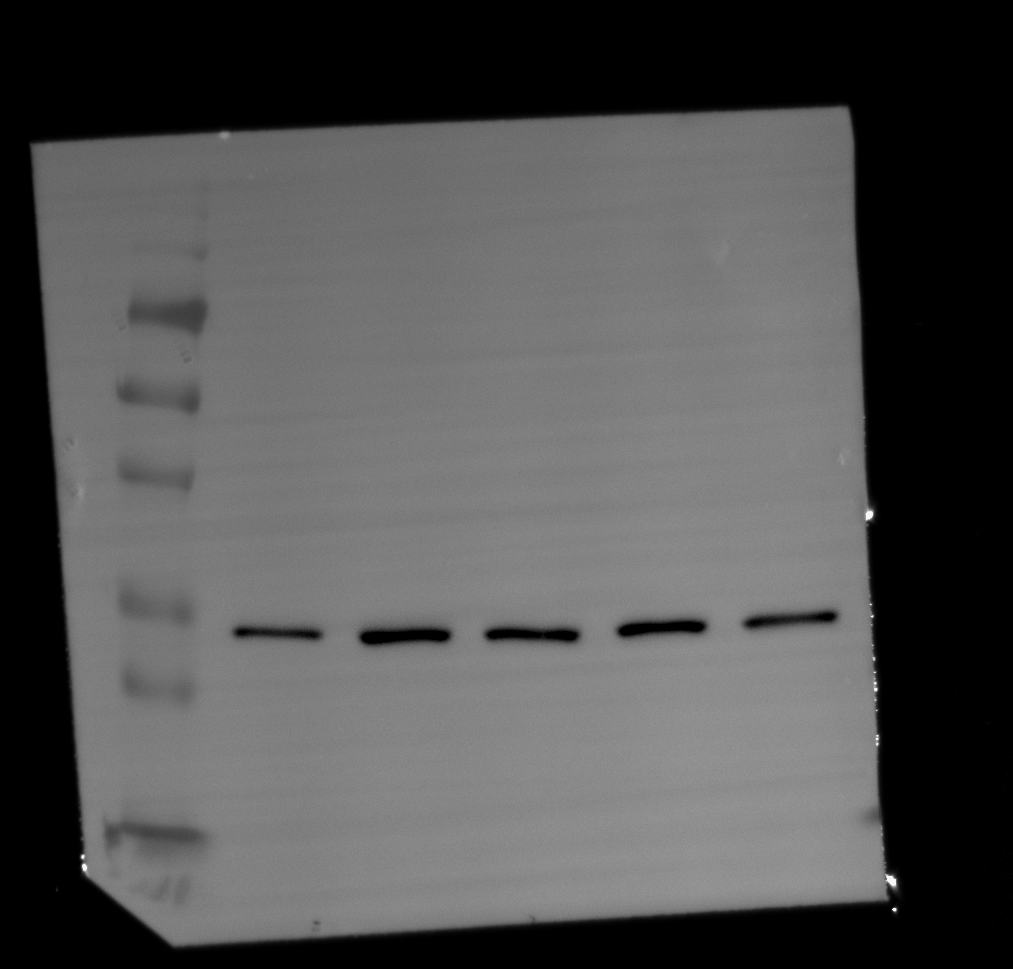

Supplement: Supplementary file 2 [file DataSheet1.zip › Western Blotting/IL-6(Ratsú⌐.tif]

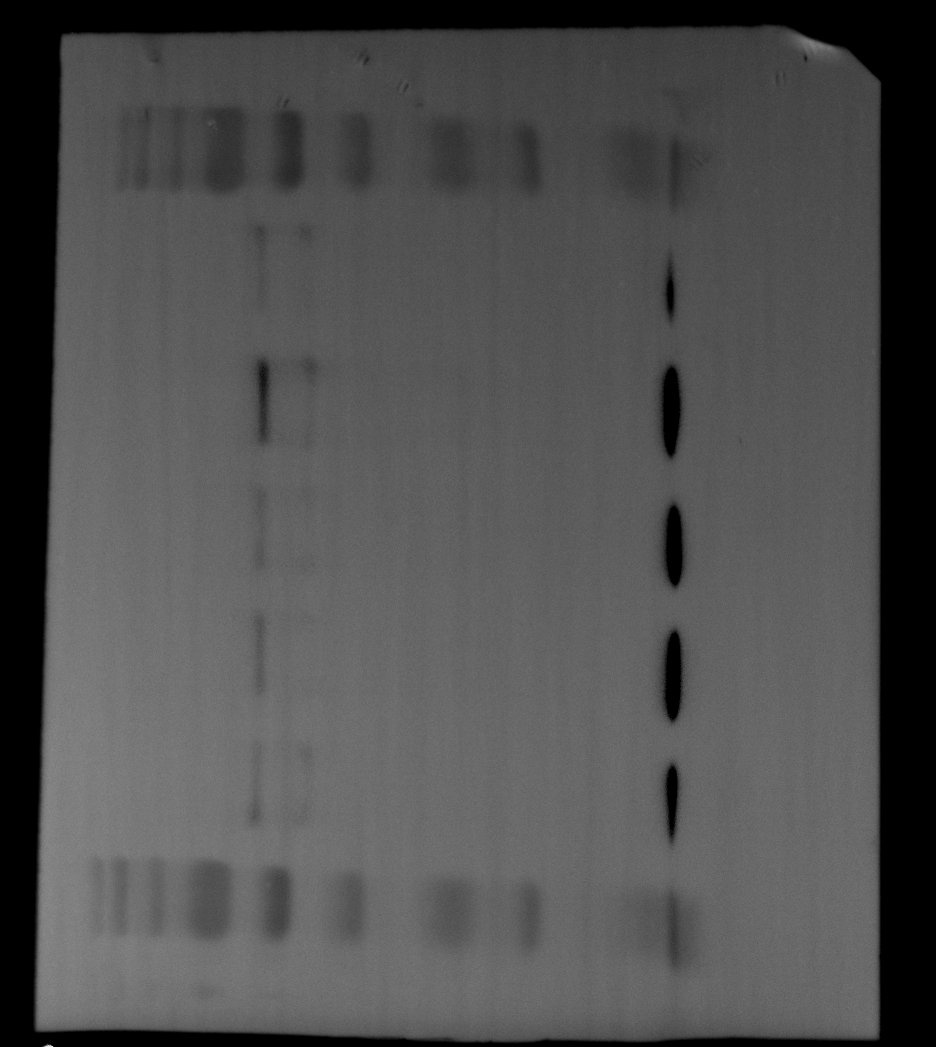

Supplement: Supplementary file 2 [file DataSheet1.zip › Western Blotting/Iba-1.tif]

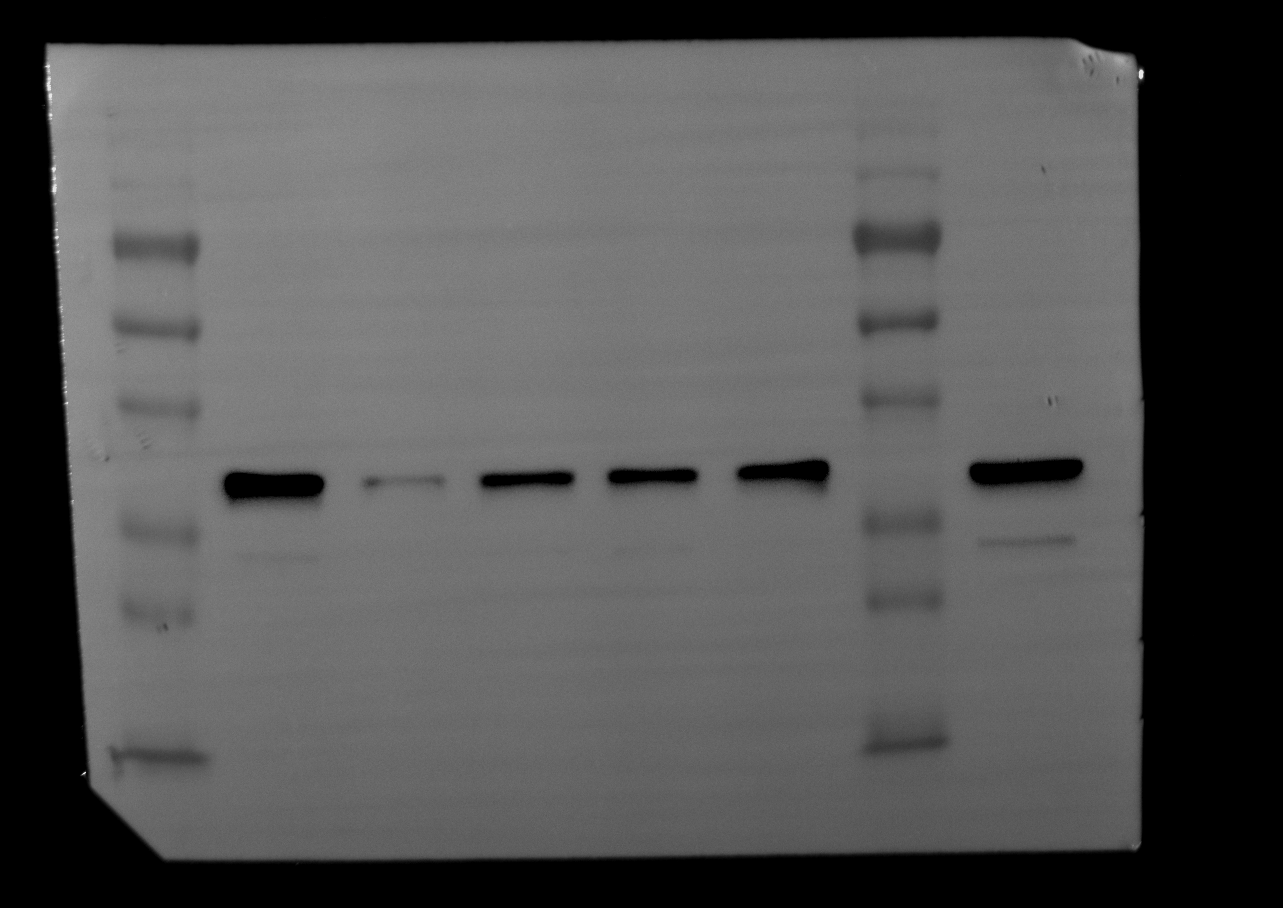

Supplement: Supplementary file 2 [file DataSheet1.zip › Western Blotting/NeuN.tif]

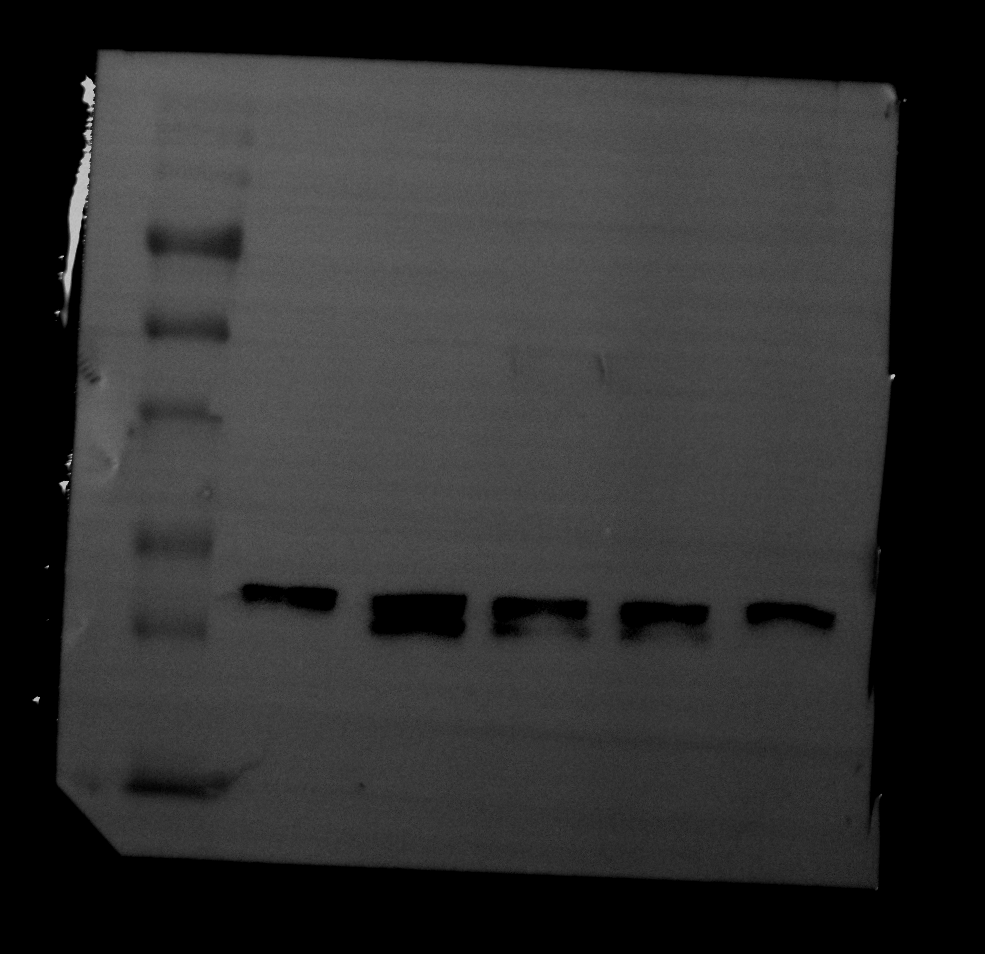

Supplement: Supplementary file 2 [file DataSheet1.zip › Western Blotting/TNF-a┴(BMECsú⌐.tif]

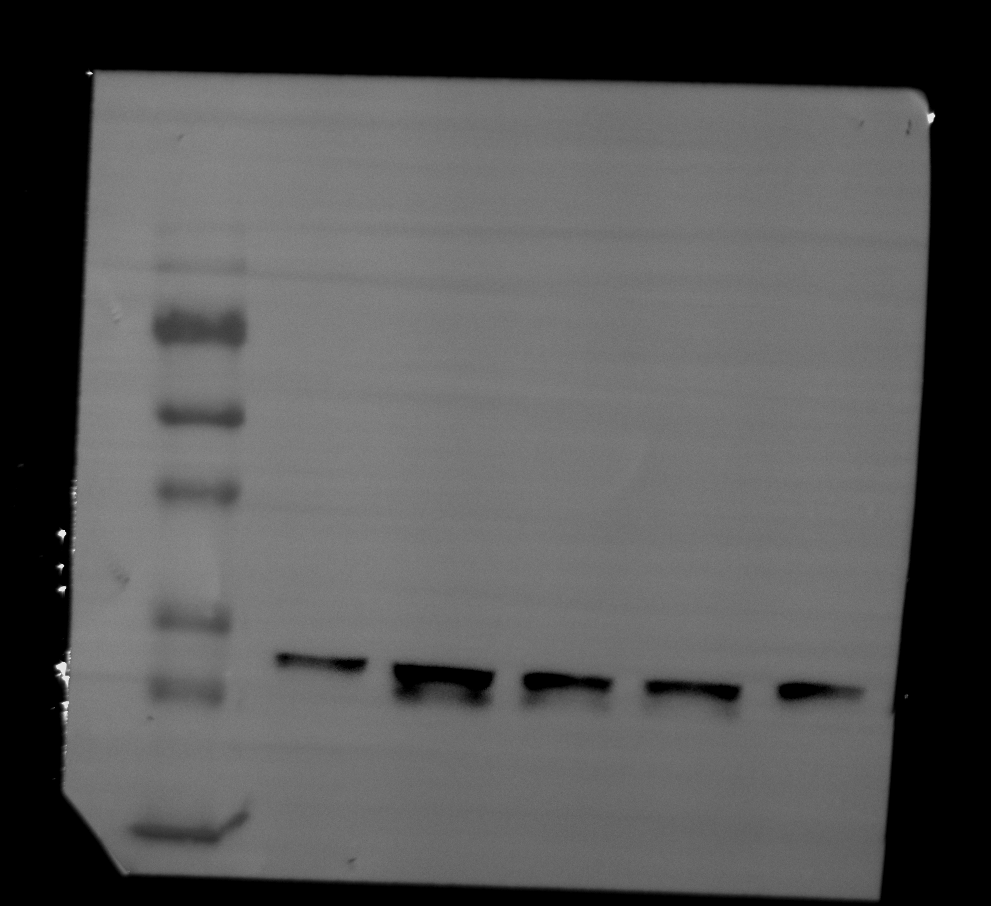

Supplement: Supplementary file 2 [file DataSheet1.zip › Western Blotting/TNF-a┴(Ratsú⌐.tif]

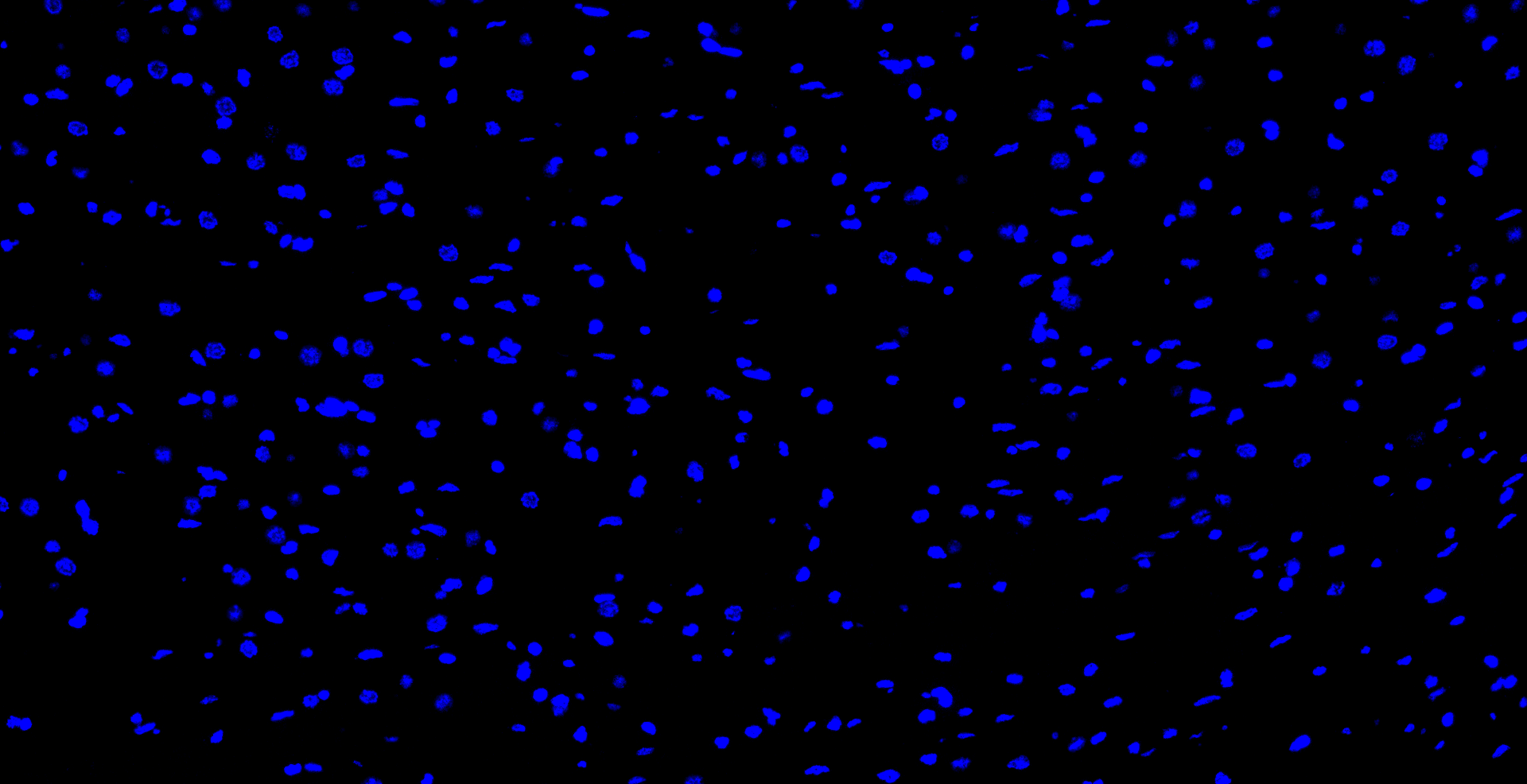

Supplement: Supplementary file 3 [file DataSheet2.zip › Immunofluorescence/MCAO(DAPI).tif]

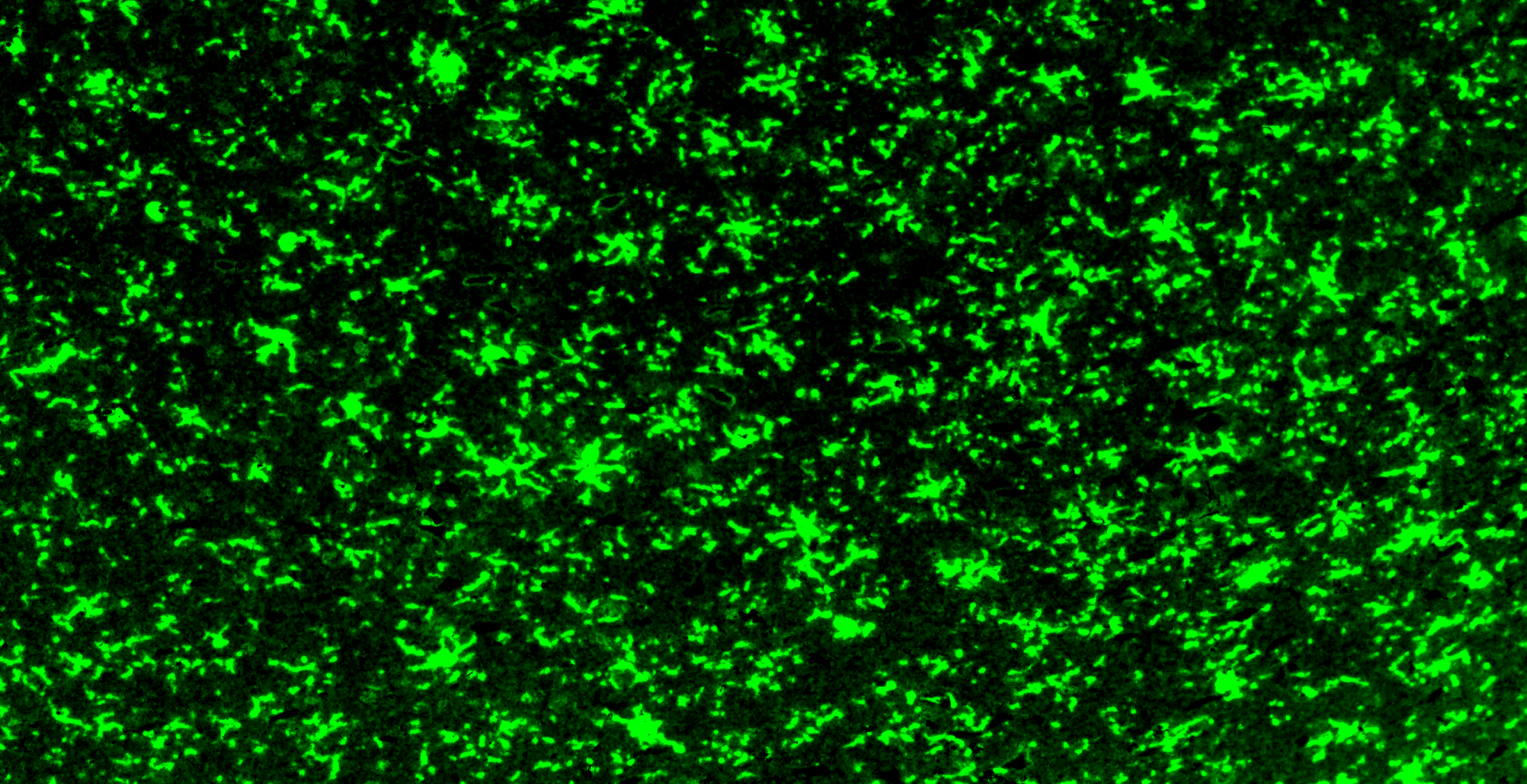

Supplement: Supplementary file 3 [file DataSheet2.zip › Immunofluorescence/MCAO(Iba-1).tif]

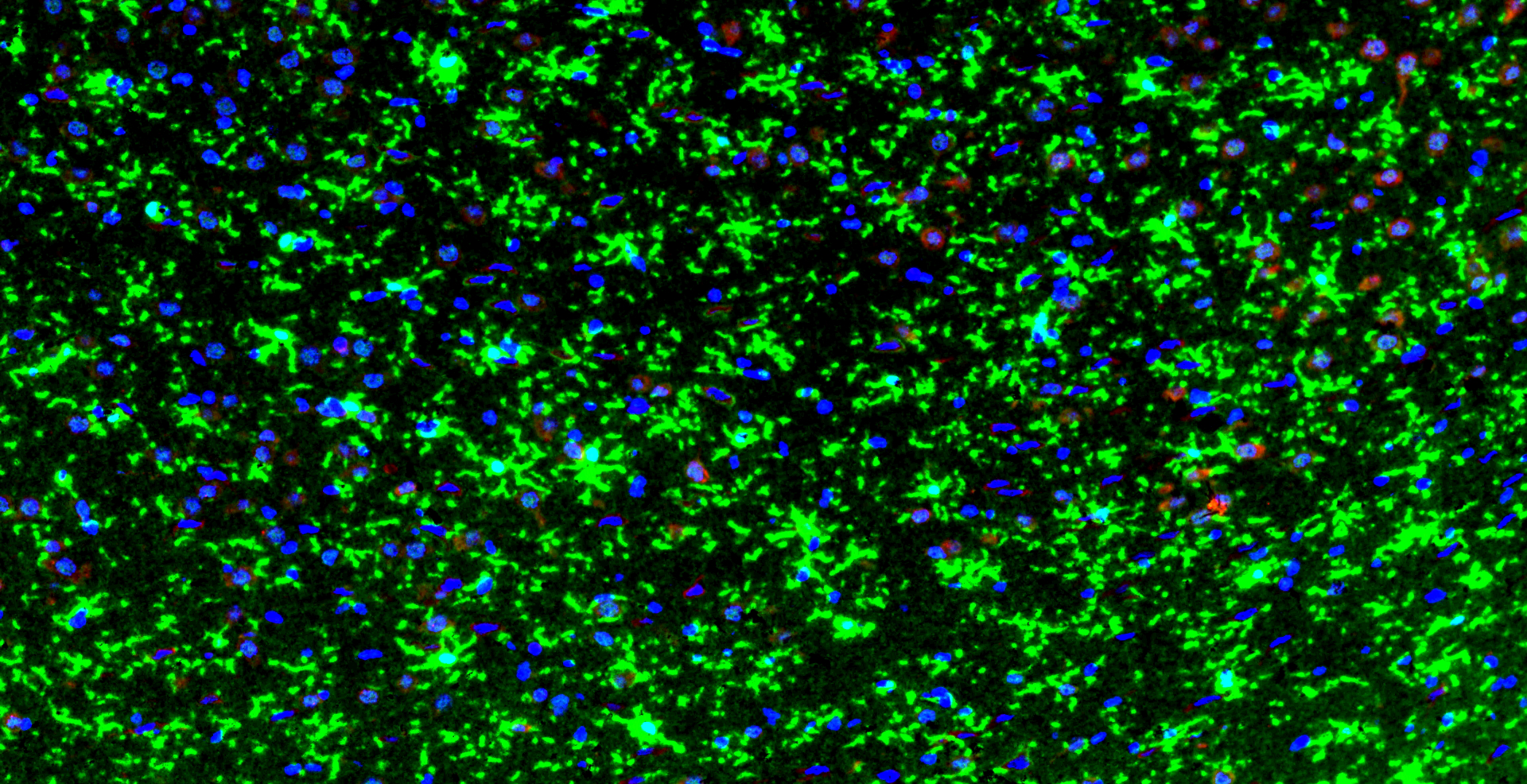

Supplement: Supplementary file 3 [file DataSheet2.zip › Immunofluorescence/MCAO(Merge).tif]

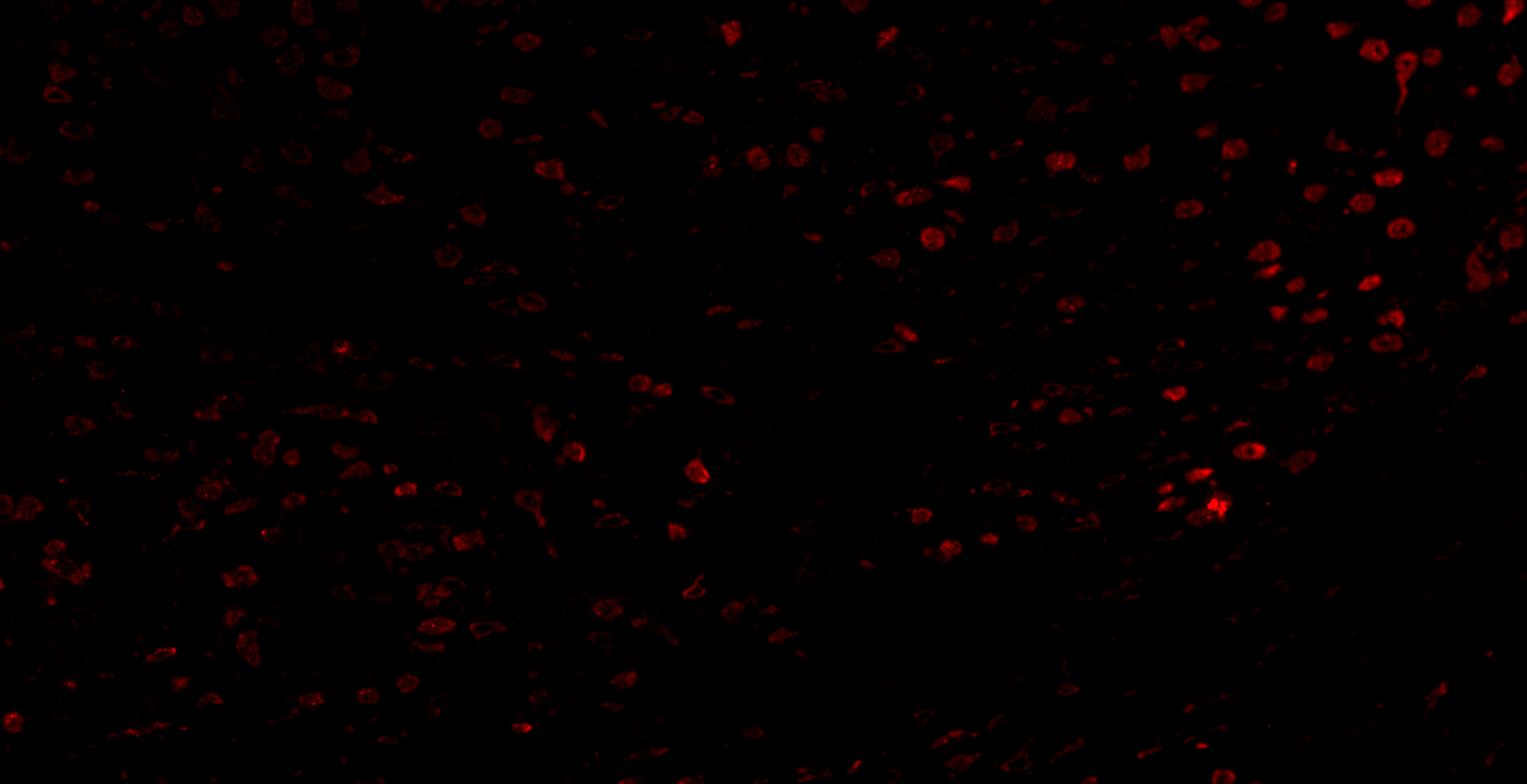

Supplement: Supplementary file 3 [file DataSheet2.zip › Immunofluorescence/MCAO(NeuN).tif]

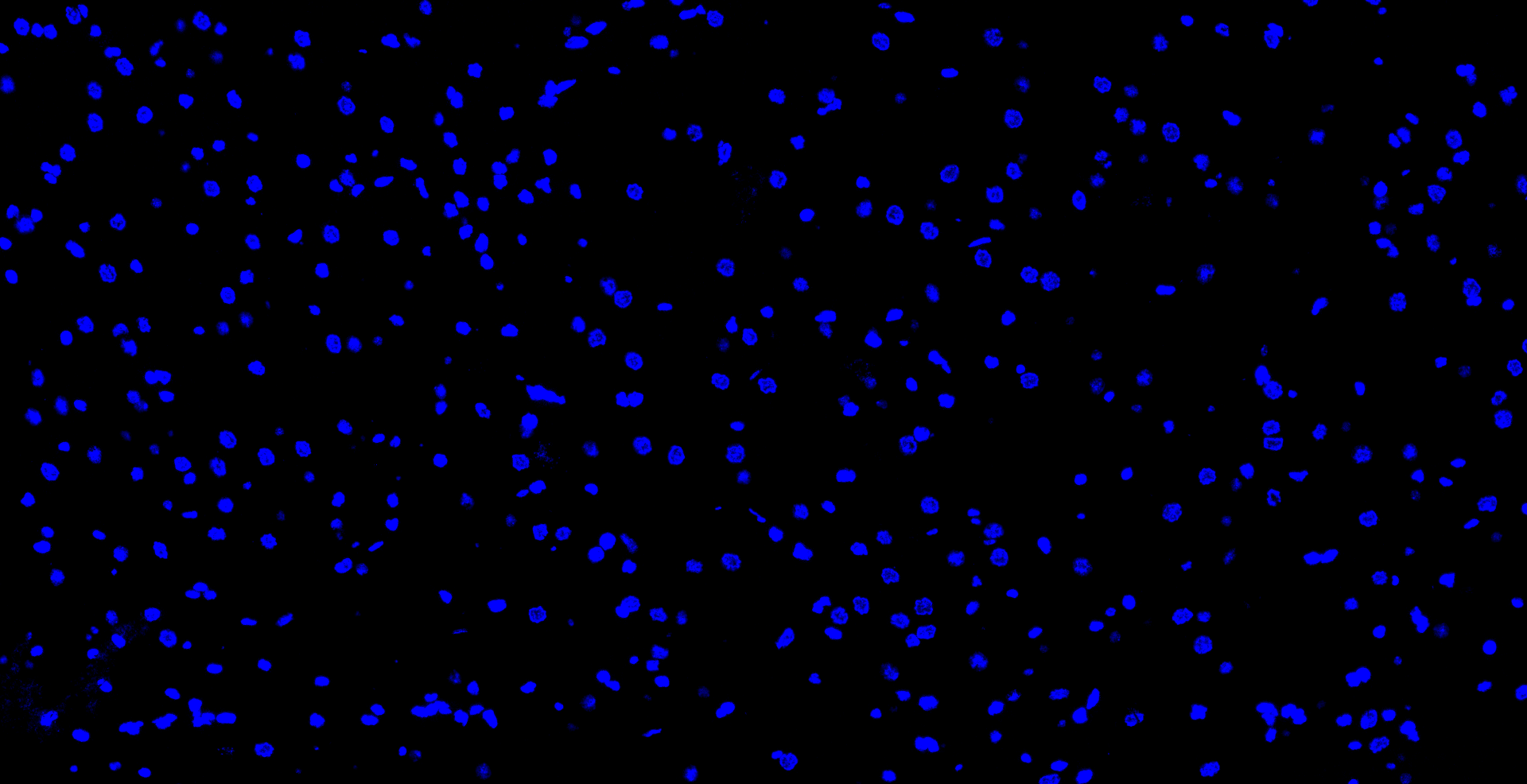

Supplement: Supplementary file 3 [file DataSheet2.zip › Immunofluorescence/MCAO+Frankincense(DAPI).tif]

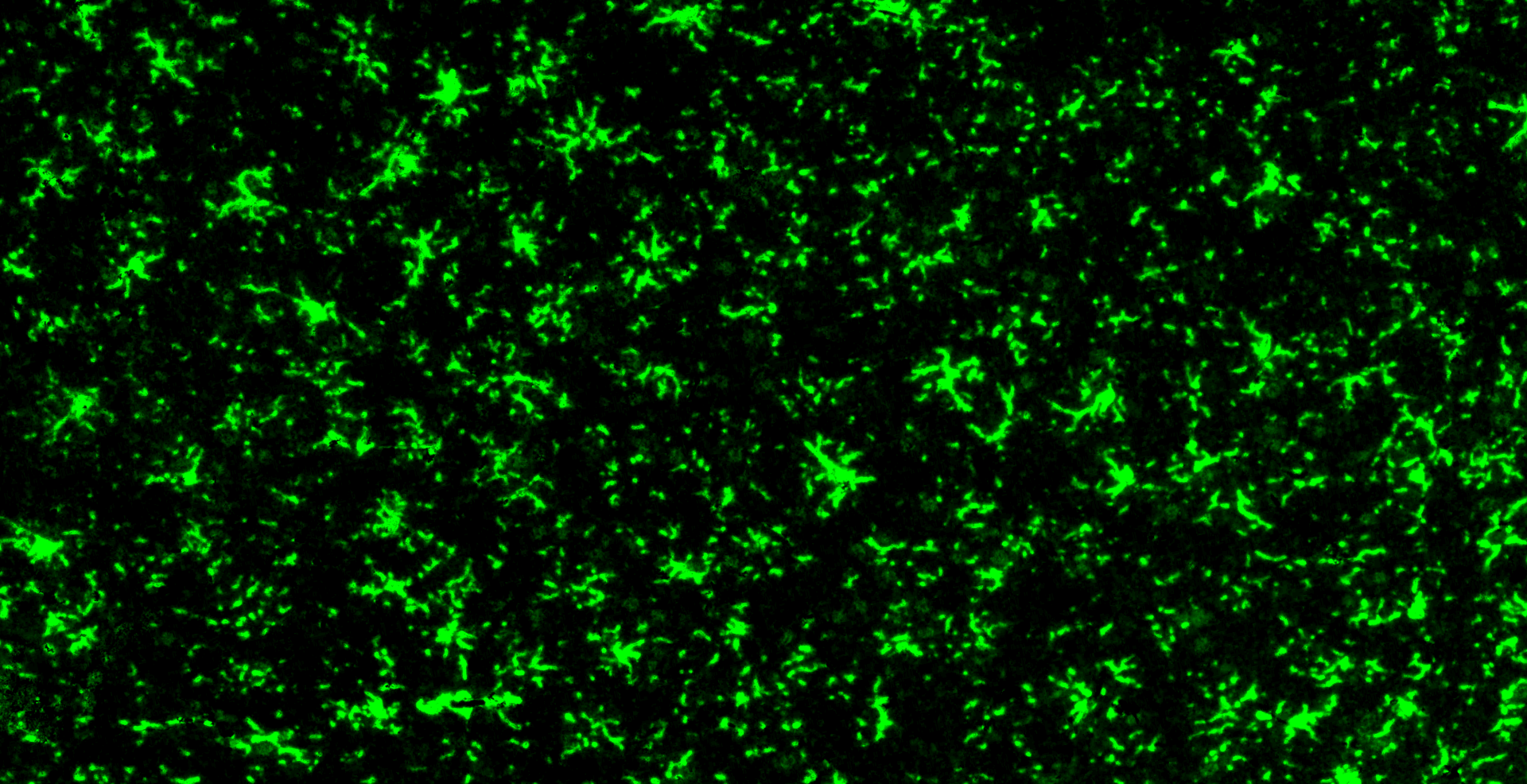

Supplement: Supplementary file 3 [file DataSheet2.zip › Immunofluorescence/MCAO+Frankincense(Iba-1).tif]

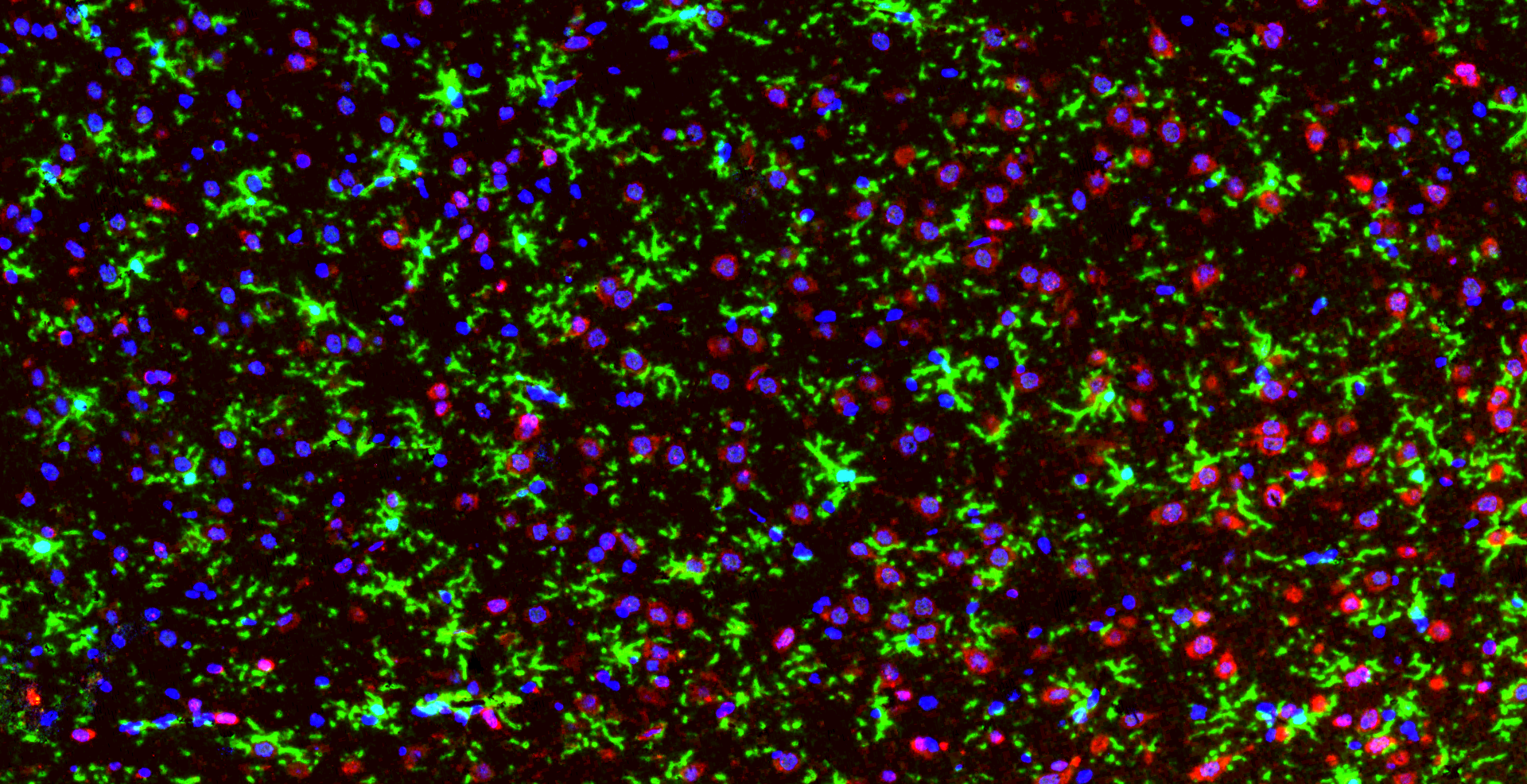

Supplement: Supplementary file 3 [file DataSheet2.zip › Immunofluorescence/MCAO+Frankincense(Merge).tif]

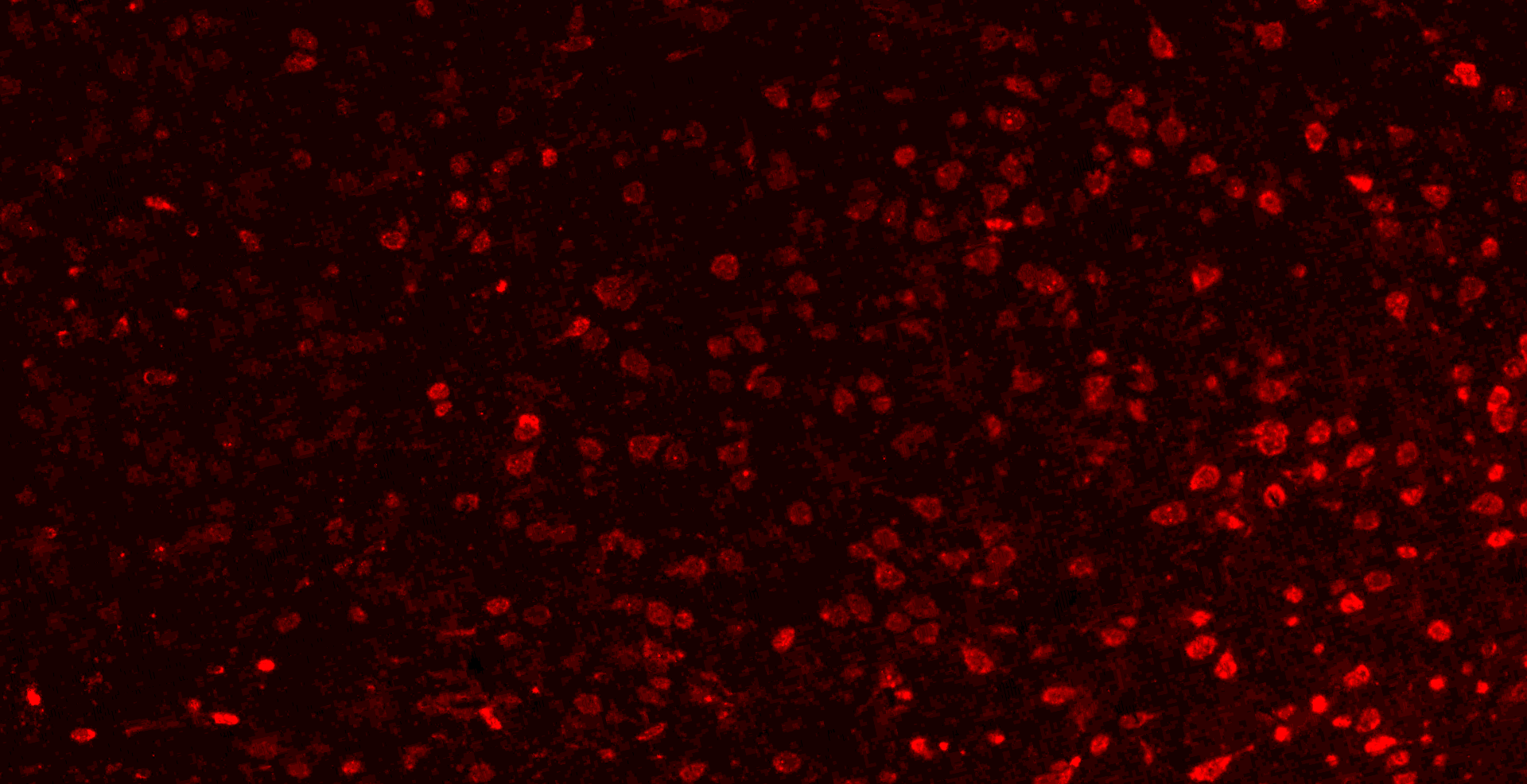

Supplement: Supplementary file 3 [file DataSheet2.zip › Immunofluorescence/MCAO+Frankincense(NeuN).tif]

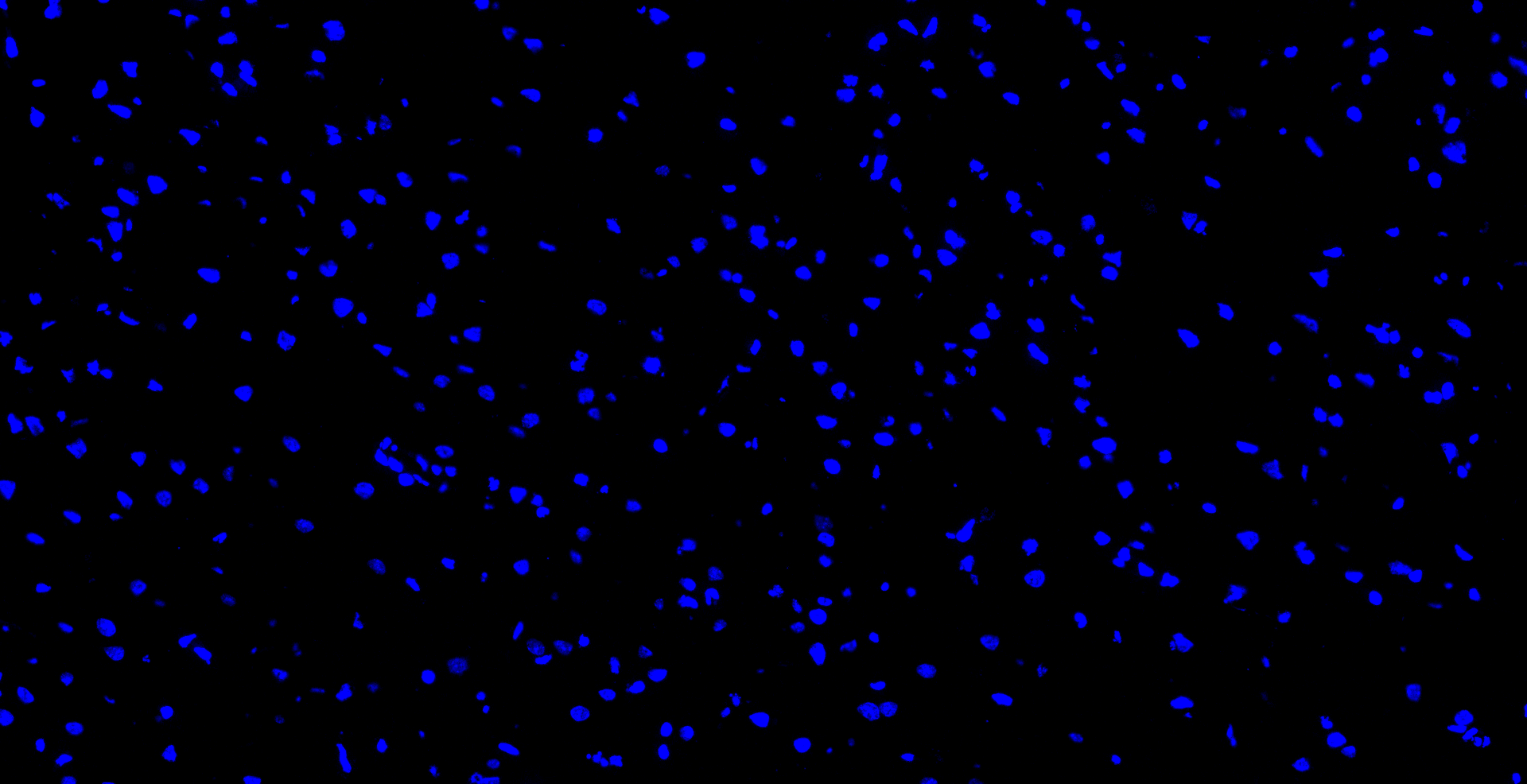

Supplement: Supplementary file 3 [file DataSheet2.zip › Immunofluorescence/MCAO+Frankincense-Myrrh(DAPI).tif]

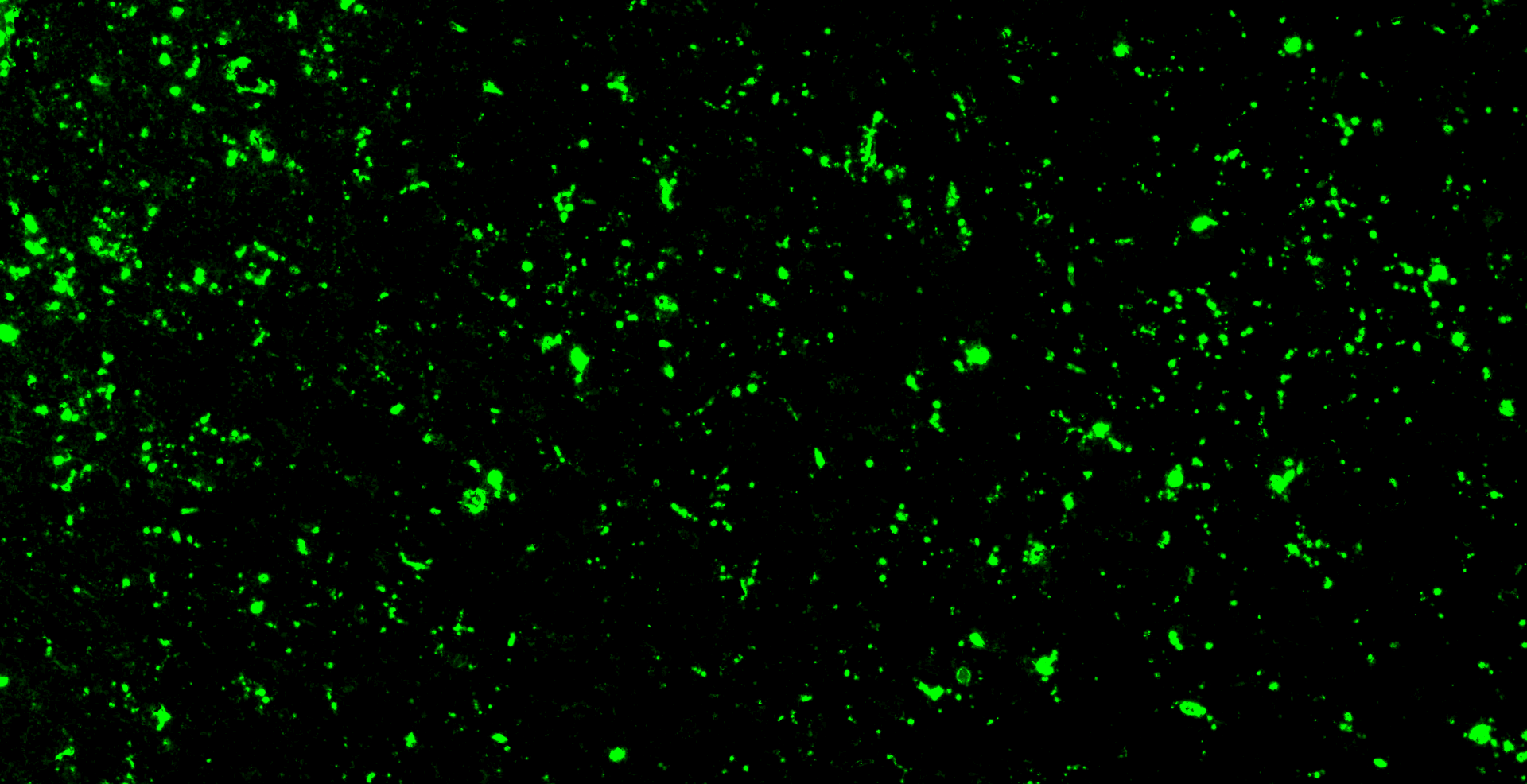

Supplement: Supplementary file 3 [file DataSheet2.zip › Immunofluorescence/MCAO+Frankincense-Myrrh(Iba-1).tif]

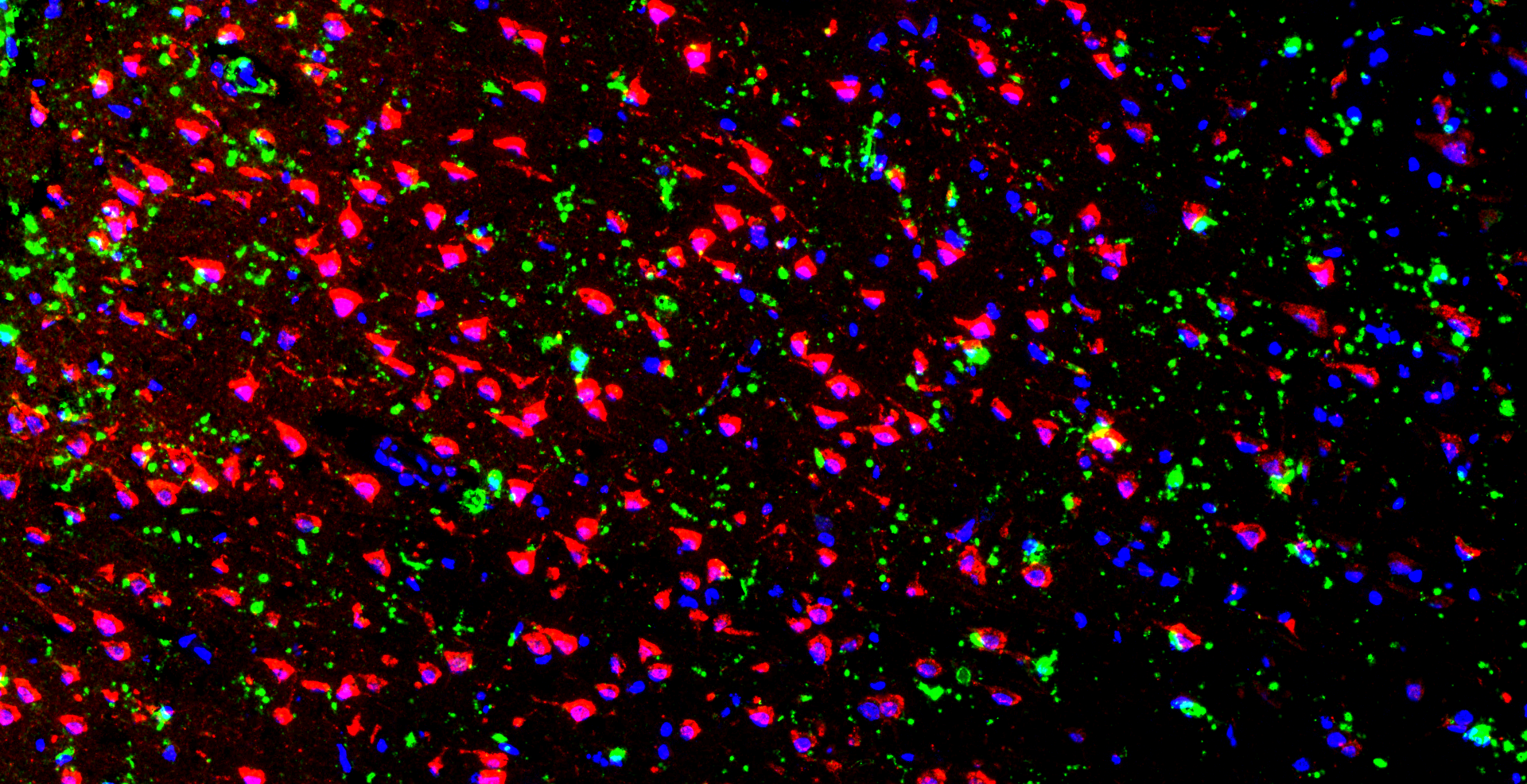

Supplement: Supplementary file 3 [file DataSheet2.zip › Immunofluorescence/MCAO+Frankincense-Myrrh(Merge).tif]

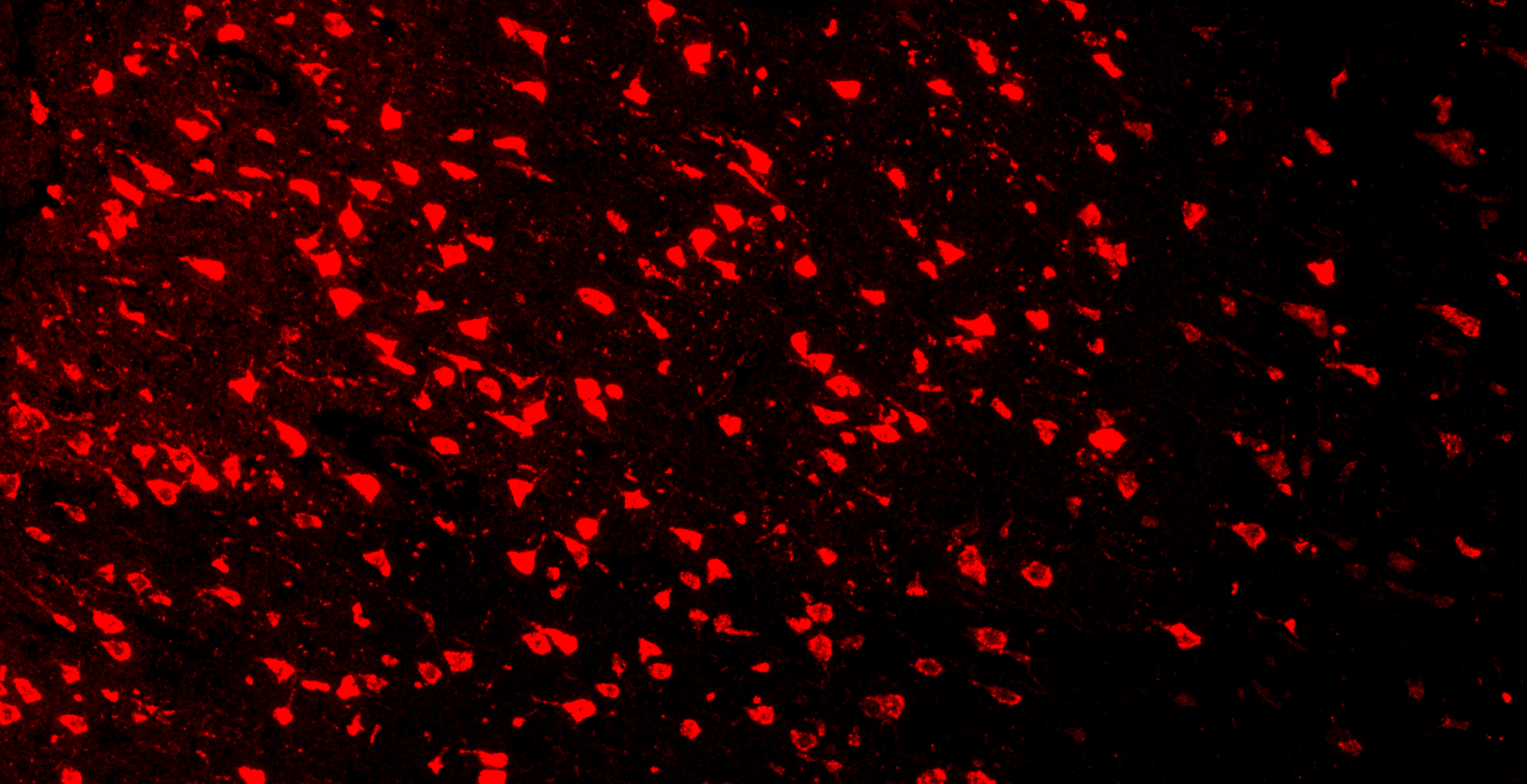

Supplement: Supplementary file 3 [file DataSheet2.zip › Immunofluorescence/MCAO+Frankincense-Myrrh(NeuN).tif]

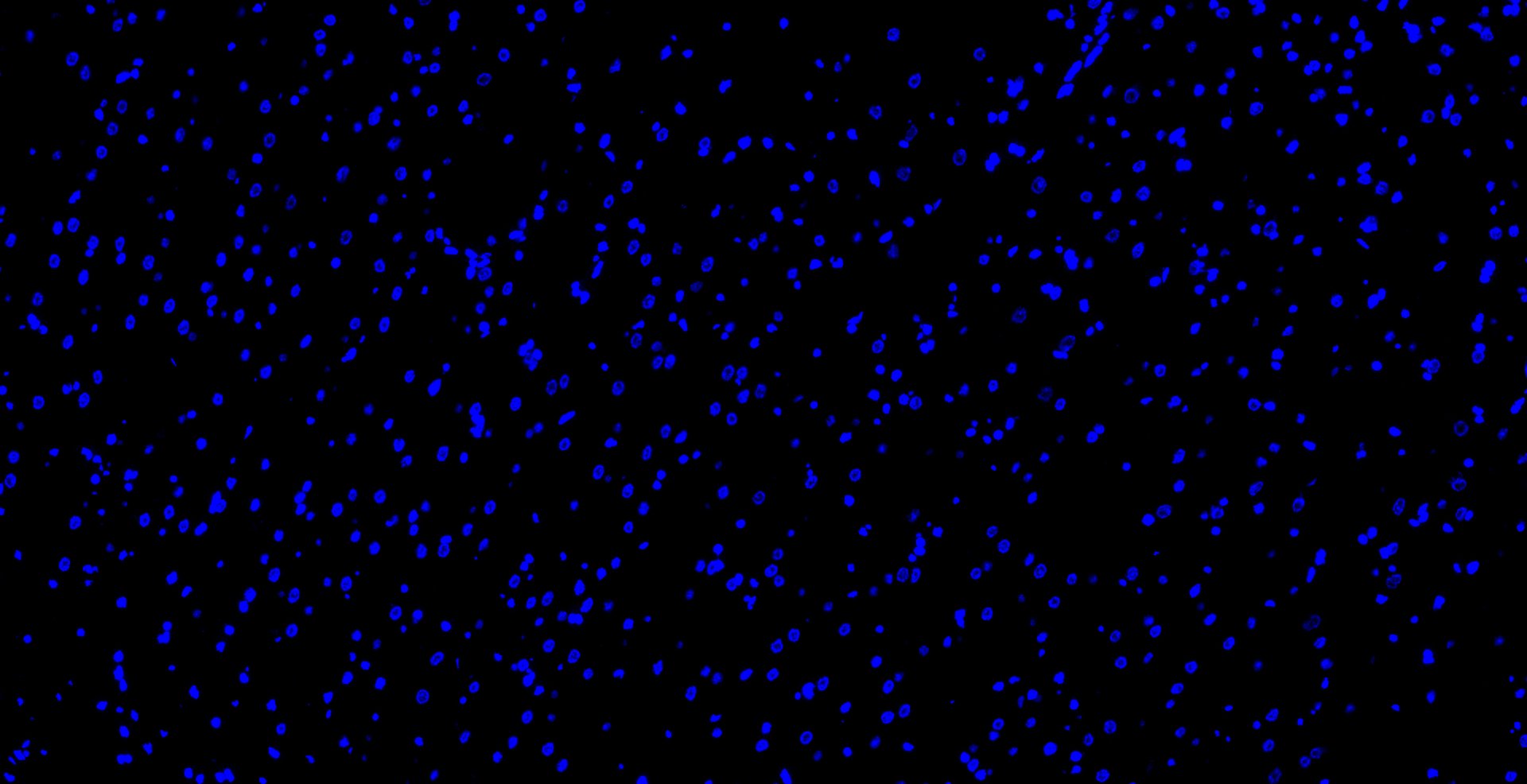

Supplement: Supplementary file 3 [file DataSheet2.zip › Immunofluorescence/MCAO+Myrrh(DAPI).tif]

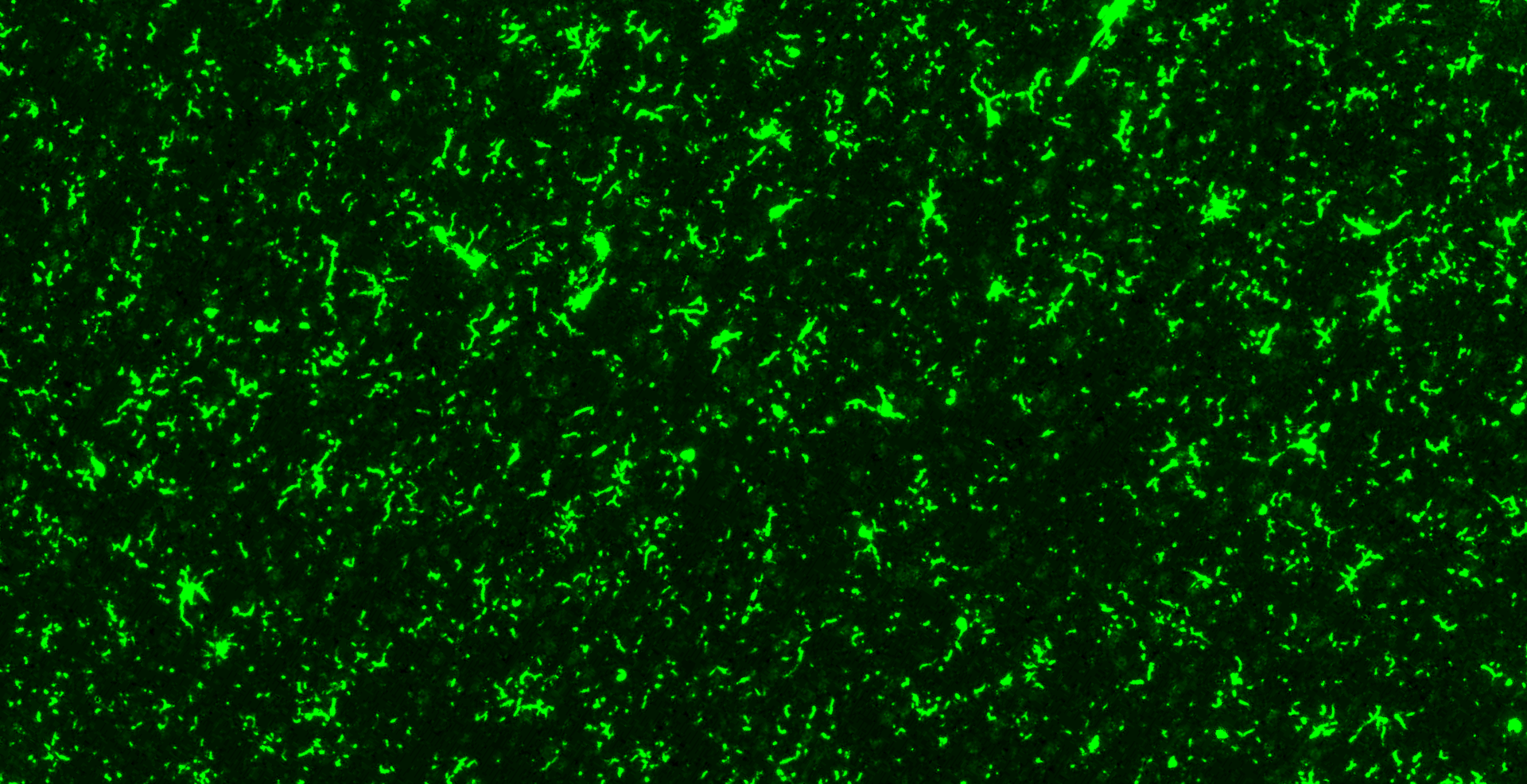

Supplement: Supplementary file 3 [file DataSheet2.zip › Immunofluorescence/MCAO+Myrrh(Iba-1).tif]

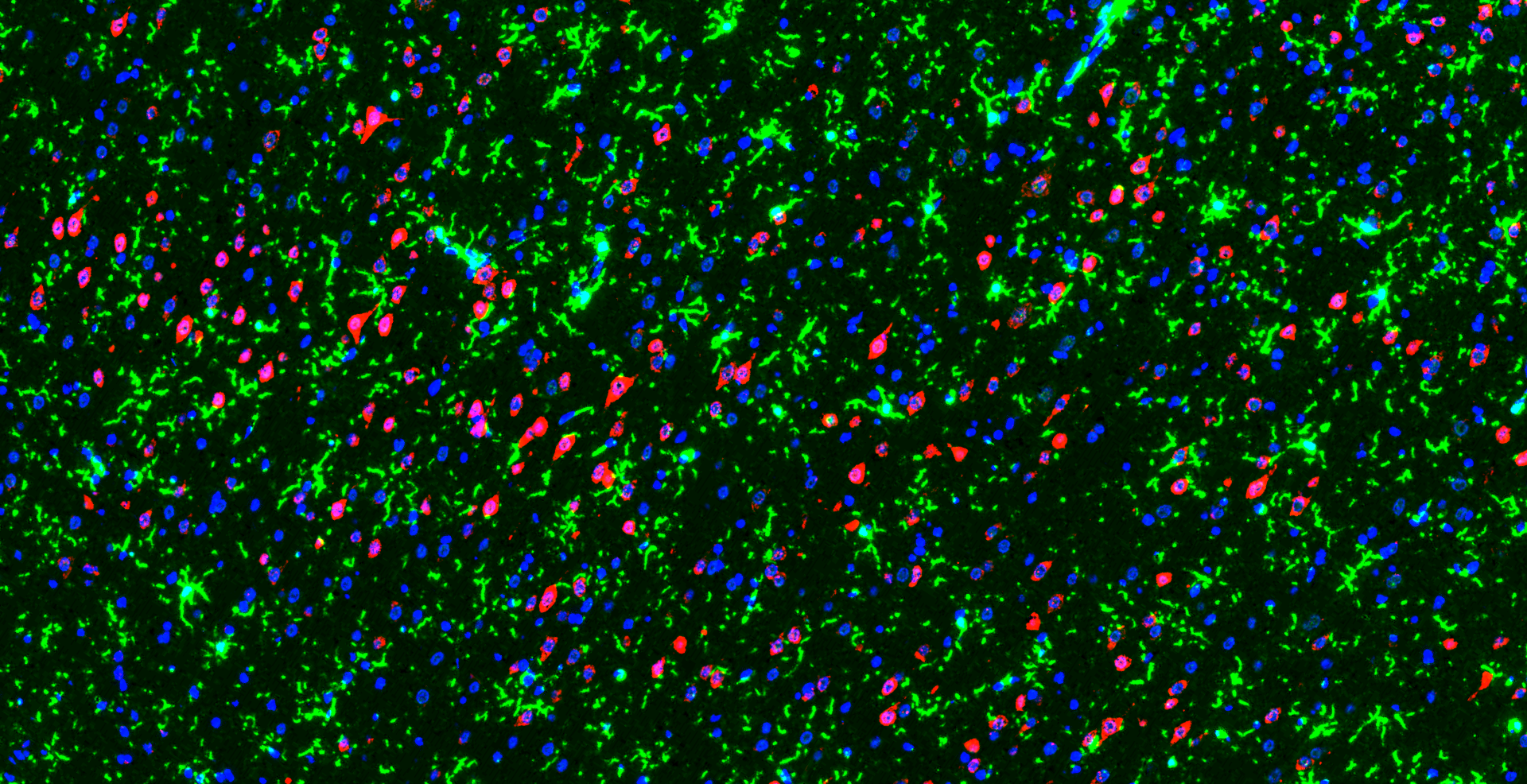

Supplement: Supplementary file 3 [file DataSheet2.zip › Immunofluorescence/MCAO+Myrrh(Merge).tif]

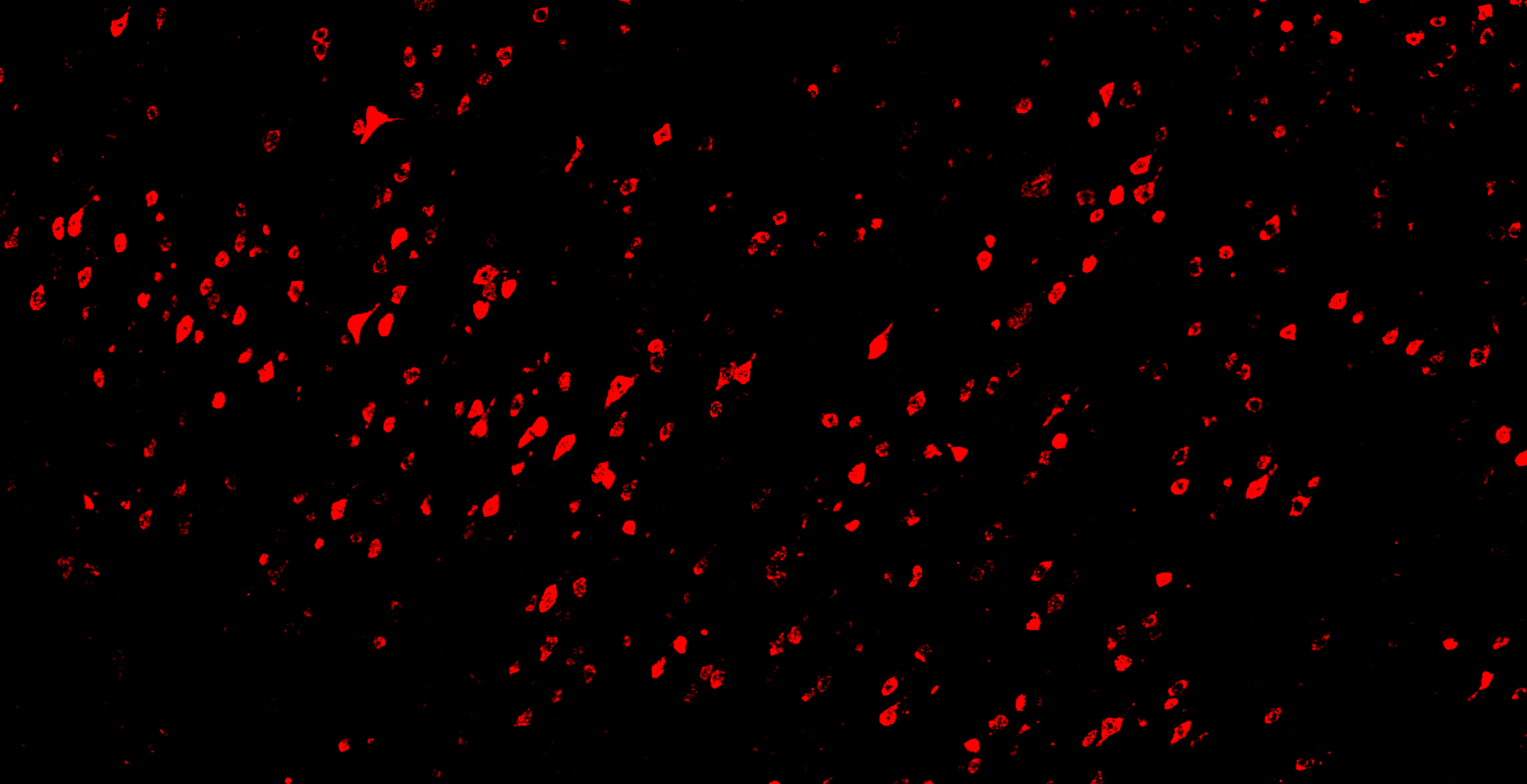

Supplement: Supplementary file 3 [file DataSheet2.zip › Immunofluorescence/MCAO+Myrrh(NeuN).tif]

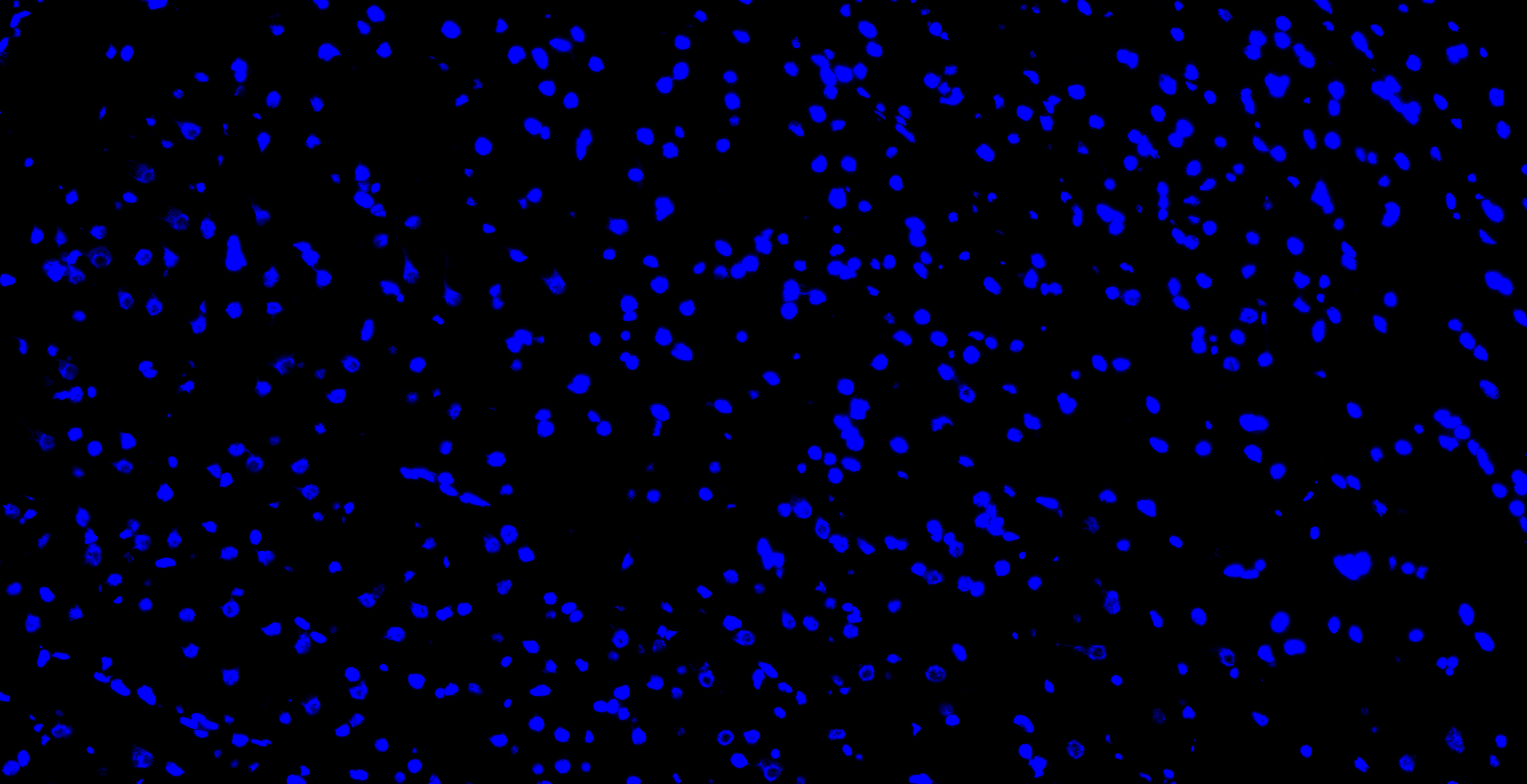

Supplement: Supplementary file 3 [file DataSheet2.zip › Immunofluorescence/Sham(DAPI).tif]

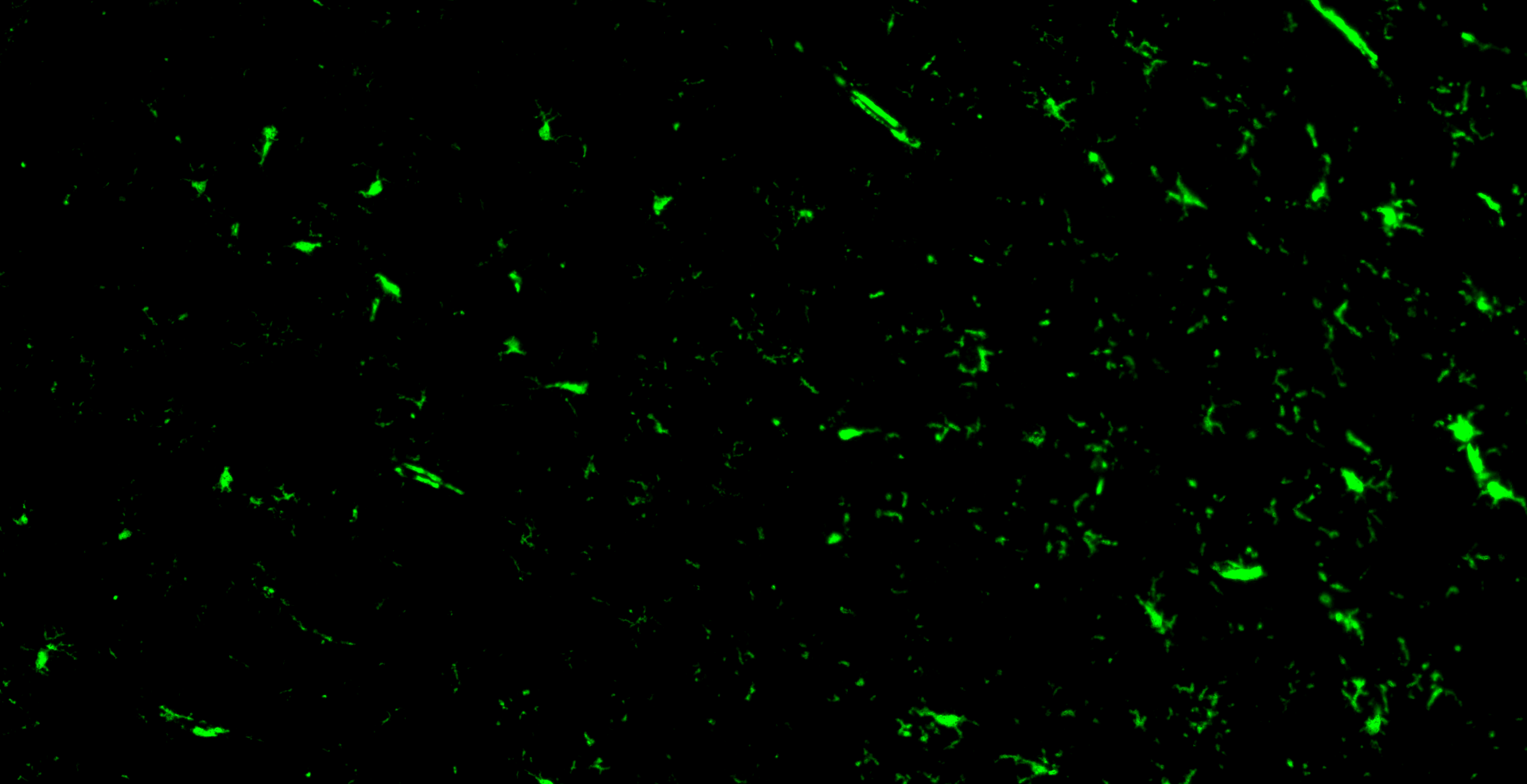

Supplement: Supplementary file 3 [file DataSheet2.zip › Immunofluorescence/Sham(Iba-1).tif]

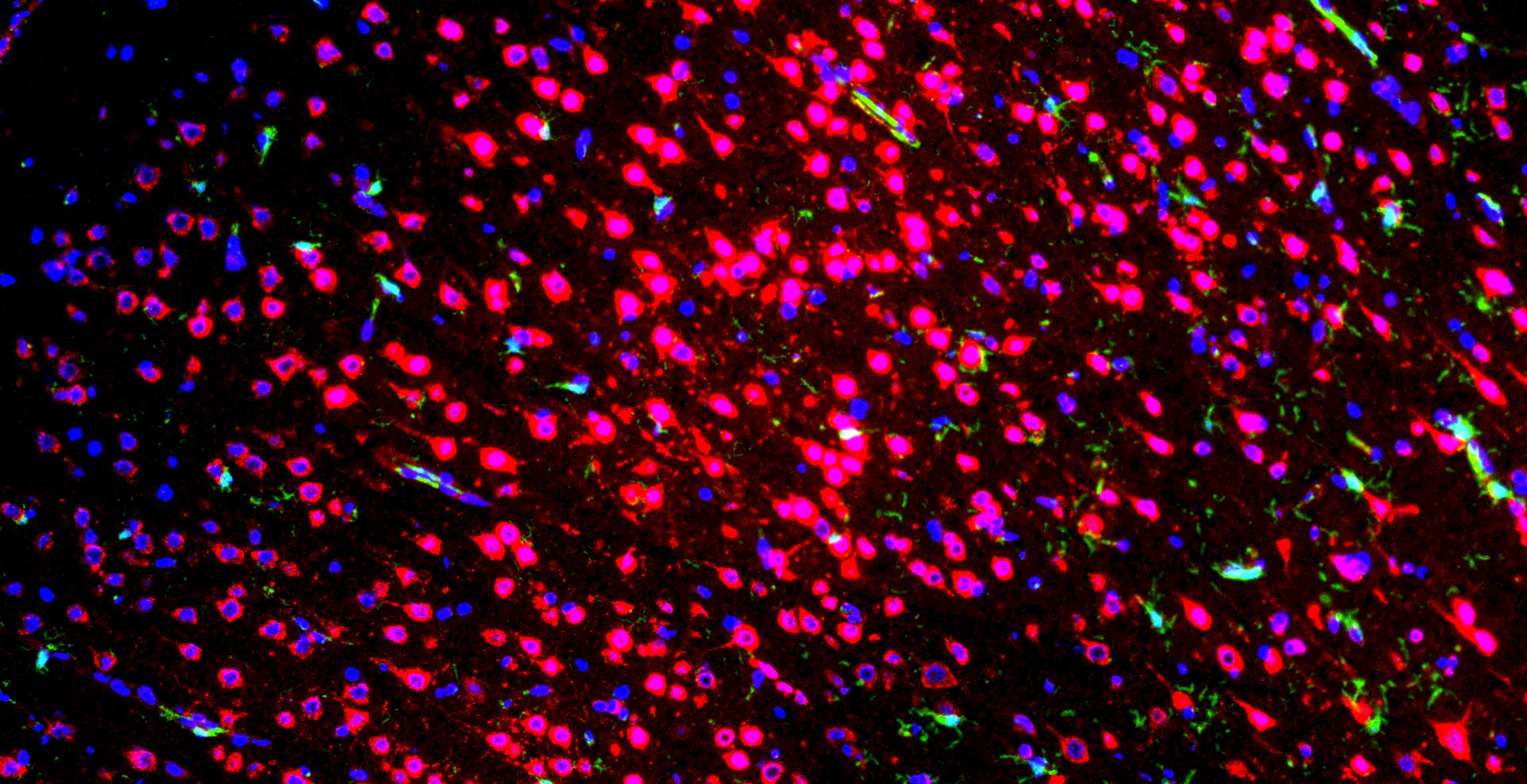

Supplement: Supplementary file 3 [file DataSheet2.zip › Immunofluorescence/Sham(Merge).tif]

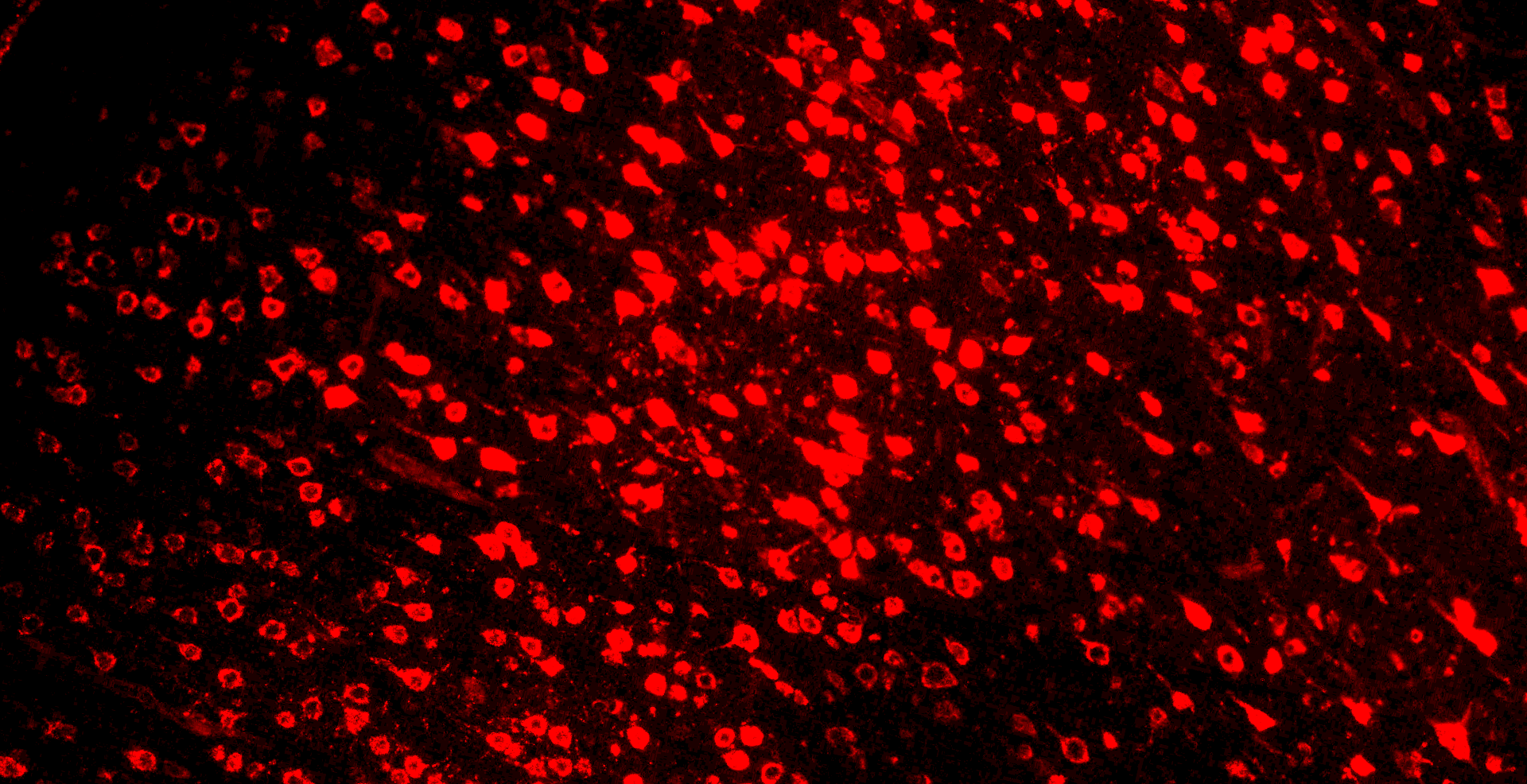

Supplement: Supplementary file 3 [file DataSheet2.zip › Immunofluorescence/Sham(NeuN).tif]
